# Supplementary material for: Reducing Calf Mortality in Ethiopia
Source: Animals (Basel). 2022 Aug 19;12(16):2126. doi: 10.3390/ani12162126 (PMC9405078; doi:10.3390/ani12162126)
Supplement: Supplementary file 1 [file animals-12-02126-s001.zip › animals-1774628-supplementary.pdf]

Supplementary Materials

The following supplementary materials accompany the paper 'Reducing calf mortality in Ethiopia'. They detail the protocols and questionnaires used in both the Epidemiological and the Intervention arms of the study.

Please note that the Young Stock Mortality Reduction Consortium (YSMRC) study included cattle, small ruminants and camels, however, the paper only includes the cattle findings. Thus, for both arms of the study, the standardized data collection forms/questionnaires include questions for sheep/goat and camel producers, as does the Physical Exam Health Scoring System for the Epidemiological arm.

For the Intervention arm, the Standard Operating Procedures (SOPs) present all cattle SOPs developed by the YSMRC, including those that were not selected for the intervention package taught to mixed crop-livestock, pastoralist and per-urban producers.

Table of Contents

Epidemiological arm 3
- Standardized data collection form 4
- Physical exam health scoring system 12

Intervention arm 16
- Questionnaires 17
  - Mixed crop-livestock 17
  - Pastoralist 20
  - Peri-urban 25
- Standard Operating Procedures (SOPs) 28
  - Mixed crop-livestock 28
  - Pastoralist 67
  - Peri-urban 95

Epidemiological arm

HOUSEHOLD ID:______________________ DATE: (dd)_____(mm)_____(yyyy)_________
YOUNG STOCK ID:___________________________ Examiner Name: _______________________

Use this form for each animal less than 6 months old that you enroll in the household. Different animals should have different forms. For animals seen for the first time, use this form. If the animal has been seen in a previous visit, fill out the Young Stock Follow Up Form. Each household should have a folder and all its forms should be kept together within the household folder.

Young Stock Enrollment Form

1) YOUNG STOCK ID ASSIGNED (Per ID protocol)	

2) Age	(days)	(Weeks)	(months)	Don't know

3) Sex (circle one)	Male	Female

4) Predominant Breed (circle one)	Local	Improved/Crossbred	Don't know

5) Name of Breed		Don't know

Animal History

For the following questions, circle one option.

6) Sire	Artificial Insemination	Live Cover		Don't know
7) Time of Birth	Day	Night		Don't know
8) Birth witnessed	Yes	No		Don't know
9) Birth location	In barn	outside	Other	Don't know
10) Type of delivery	normal	assisted		Don't know
11) Colostrum fed within 24 hours?	Yes	No		Don't know
12) If answer to question 11 is no, was this because				
Calf alert but unable to suck	Calf depressed, unable to suck	Other		Don't know
13) Navel dipped	Yes	No		Don't know
14) Did animal have continuous access to dam for the first 24 hours of life?	Yes	No		Don't know
15) Separate housing from herd?	Yes	No		Don't know
16) Animal housed with dam?	Yes	No		Don't know
17) Animal housed with other young stock?	Yes	No		Don't know

18) Any vaccinations given to animal since birth?	Yes	No	Don't know
19) If yes, what type of vaccines?			

HOUSEHOLD ID:______________________ DATE: (dd)_____(mm)_____(yyyy)_________
YOUNG STOCK ID:___________________________ Examiner Name: _______________________

20) Any medications given to animal since birth?	Yes	No	Don't know
21) If yes to question 20, what type of medications? Mark from the following below			
Dewormers	Yes	No	Don't know
Vitamins	Yes	No	Don't know
Oral electrolytes	Yes	No	Don't know
Other, specify	Yes	No	Don't know

22) Has this animal shown any signs of illness since birth?	Yes	No	Don't know

23) If yes to question 22, mark what symptoms of illness has been observed			
Nasal discharge	Yes	No	Don't know
Cough	Yes	No	Don't know
Difficulty breathing	Yes	No	Don't know
Ocular discharge	Yes	No	Don't know
Cloudy eyes	Yes	No	Don't know
Head tilt	Yes	No	Don't know
Circling	Yes	No	Don't know
Not suckling	Yes	No	Don't know
Diarrhea (not bloody)	Yes	No	Don't know
Diarrhea (bloody)	Yes	No	Don't know
Bloat	Yes	No	Don't know
Lameness	Yes	No	Don't know
Swollen joints	Yes	No	Don't know
Umbilical infections or swelling	Yes	No	Don't know
Difficulty urinating	Yes	No	Don't know
Straining to defecate	Yes	No	Don't know
Birth defects	Yes	No	Don't know
Collapse	Yes	No	Don't know
Predation	Yes	No	Don't know
Trauma	Yes	No	Don't know
Bleeding from orifices	Yes	No	Don't know
Other, describe			

HOUSEHOLD ID:______________________ DATE: (dd)_____(mm)_____(yyyy)_________
YOUNG STOCK ID:___________________________ Examiner Name: _______________________

24) Have any of the following procedures been done to this animal?			
Castration	Yes	No	Don't know
Dehorning	Yes	No	Don't know
Branding	Yes	No	Don't know
Ear notch	Yes	No	Don't know
Tail docking	Yes	No	Don't know

Animal Feeding History

For the following questions, circle one option

25) Is animal fed milk?	Yes, MILK	Yes, MILK REPLACER	Not fed Milk or Milk replacer	Don't know
**If animal is fed either milk or milk replacer, answer the following**				
27) How often is animal fed milk/milk replacer?	Once a day	Twice a day	More than twice a day	Don't know
28) At each feeding, how much milk is fed?		(Liters)		Don't know

29) Is animal fed supplemental food?		Yes	No	Don't know
If yes to supplemental food				
30) How often is fed supplemental food	Once a day	Twice a day	More than twice a day	Don't know

31) Is animal given water?		Yes	No	Don't know
If yes to water				
32) How often is the animal given water?	Once a day	Twice a day	More than twice a day	Don't know
33) Does animal have its own water trough?		Yes	No	Don't know

34) Is the animal weaned?	Yes	No	Don't know
If yes to weaned			
35) What age was animal weaned at?		(months)	Don't know

Animal Environment

Inspect animal housing place. Look at where this animal is housed and answer the following questions.

36) Is animal housed on bedding?	Yes	No	Don't know
37) Does the area where the animal lies down look clean and dry?	Yes	No	Don't know
38) If all the animals housed together were going to stand up at the same time, would they have enough room to do so and turn around without touching each other?	Yes	No	Don't know

HOUSEHOLD ID:______________________ DATE: (dd)_____(mm)_____(yyyy)_________
YOUNG STOCK ID:___________________________ Examiner Name: _______________________

Dam History

For the following questions, circle one option

39) Age of dam at parturition	(years)		Don't know
40) Dam gestation number			Don't know
41) Were any vaccines given to the dam within 2 months of delivery	Yes	No	Don't know
42) If yes to vaccines, specify type			

43) Is dam alive?	Yes	No	Don't know
If yes, answer the following			
44) Dam Body Condition Score (cows/sheep/goats use scale 1 -5; camels use scale 0-5)			Don't know
45) Dam milk production (include all milk, i.e., milk given to calf and milk sold /human consumption)		(Liters/day)	Don't know
46) Is dam mothering instinct adequate?	Yes	No	Don't know

If no to question #43, answer the following			
47) Did the dam die within the first 2 weeks after parturition?	Yes	No	Don't know

HOUSEHOLD ID:______________________ DATE: (dd)_____(mm)_____(yyyy)_________
YOUNG STOCK ID:___________________________ Examiner Name: _______________________

POST - WEANING ADDENDUM – only fill in this section if the animal has already been weaned

Type of feed and quantity given **after** weaning. Please specify type and quantity per animal:
RAINY SEASON

	**Calves**	**Camel Calves**	**Lambs**	**Kids**
48) Grazing (hours of grazing)				
49) Legumes (Alfalfa, Sesbania, Leucaena) mixed with roughage/grazing. Specify type and quantity				
50) Concentrates, type and quantity				
51) Hay, type and quantity				
52) Urea treated straw, quantity				
53) Mineral blocks				
54) Others, type and quantity				
55) Amount of hours animals have access to water (1 time/day, 2 times/day, 3 or more times/day)				

HOUSEHOLD ID:______________________ DATE: (dd)_____(mm)_____(yyyy)_________
YOUNG STOCK ID:___________________________ Examiner Name: _______________________

DRY SEASON

	**Calves**	**Camel Calves**	**Lambs**	**Kids**
56) Grazing (hours of grazing)				
57) Legumes (Alfalfa, Sesbania, Leucaena) mixed with roughage/grazing. Specify type and quantity				
58) Concentrates, type and quantity				
59) Hay, type and quantity				
60) Urea treated straw, quantity				
61) Mineral blocks				
62) Others, type and quantity				
63) Amount of hours animals have access to water (1 time/day, 2 times/day, 3 or more times/day)				

HOUSEHOLD ID:______________________ DATE: (dd)_____(mm)_____(yyyy)_________
YOUNG STOCK ID:____________________________ Examiner Name: ________________________

Physical Exam Form

Before restraining the animal, make observations about general attitude.

1) **Attitude (circle one)**	Bright/Alert	Depressed	Lethargic	Comatose

2) **General notes if necessary (emaciation, in pain, etc.)**

Once you are able to restrain the animal, record the following:

3) **Rectal Temperature**		Celsius
Use rectal temperature key to give a temperature score below		

37.7 - 38.2 = 0
38.3 - 38.8 = 1
38.9 - 39.3 = 2
>39.3 = 3

4) Respiratory Score – USE SCORING CARD:

**Temperature Score (°C)** (0-3)	**Cough Score** (0-3)	**Nasal Discharge Score** (0-3)	**Eye Score** (0-3)	**Ear Scoe** (0-3)	**Increased Respiratory effort?** 0 – no 1 - yes

Thoracic Auscultation:

5) **Respiratory Rate**		Breaths per minute	
Are wheezes present?	Yes	No	Don't know
Are crackles present?	Yes	No	Don't know
Are lung sounds muffled?	Yes	No	Don't know
Is animal coughing?	Yes	No	Don't know
6) **Heart Rate**		Beats per minute	
Are there any abnormal heart sounds?	Yes	No	Don't know
7) **Notes on Thoracic Auscultation**			

Abdominal Auscultation:

8) **Ruminal Contractions**		Number per minute	
Are peristaltic sounds increased?	Yes	No	Don't know
Are there any ping sounds?	Yes	No	Don't know
9) **Notes on Abdominal Auscultation**			

HOUSEHOLD ID:______________________ DATE: (dd)_____(mm)_____(yyyy)_________
YOUNG STOCK ID:___________________________ Examiner Name: _______________________

10) Body Condition Score<br>(1-5 for calves/kids/goats ; 0-5 for camels)			
11) Umbilicus Examination<br>(Use thumb as reference)	Normal	Abnormal	Don't know
12) Notes on umbilicus exam			
13) Limb Joints Examination	Normal	Abnormal<br>(Enlarged, painful, fluid accumulation)	Don't know
14) Notes on joints exam			

15) Fecal Score - USE SCORING CARD (circle one)

NORMAL	Pasty, normal color	Loose	Watery
0	1	2	3

16) Blood in stool?	Yes	No	Don't know
17) Notes on fecal exam			

18) Samples Collected (circle all that apply)

Sample Type				Guidance	
				Who?	STORAGE
1	Blood, EDTA tube	Yes	No	ALL	Cooler
2	Blood, Red top tube	Yes	No	ALL	Cooler
3	Feces, plain	Yes	No	ALL	Cooler
4	Feces, VTM	Yes	No	ALL	LN2
5	Feces, Amies	Yes	No	ALL	Cooler
6	Deep Nasal Swab, Amies	Yes	No	Large Calves	Cooler
7	Rostral nasal swab, VTM	Yes	No	ALL	LN2
8	Rostral nasal swab, Amies	Yes	No	Small ruminants, small calves	Cooler
9	Colostrum/Milk	Yes	No	Dams within 7 days parturition	LN2

Field Sample Collection Reference Documents for Young Stock Enrollment Physical Exam Form

https://fyi.extension.wisc.edu/heifermgmt/files/2015/02/calf_health_scoring_chart.pdf

SCHOOL OF VETERINARY MEDICINE
University of Wisconsin-Madison

Calf Health Scoring Criteria

0	1	2	3
**Rectal temperature**			
37.7-38.2 C	38.3-38.8 C	38.9-39.3 C	>39.3 C
**Cough**			
None	Induce single cough	Induced repeated coughs or occasional spontaneous cough	Repeated spontaneous coughs
**Nasal discharge**			
Normal serous discharge	Small amount of unilateral cloudy discharge	Bilateral, cloudy or excessive mucus discharge	Copious bilateral mucopurulent discharge
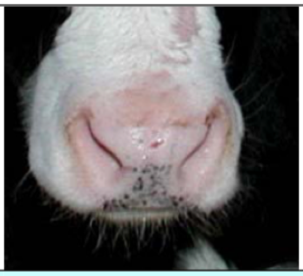	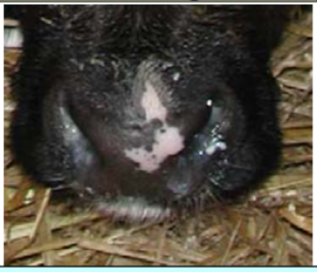	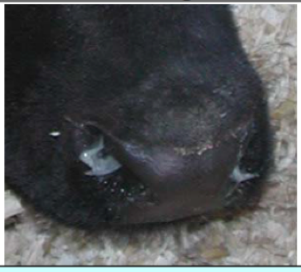	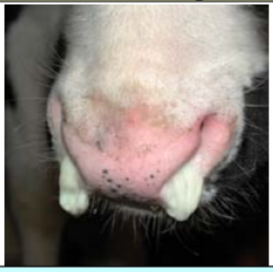
**Eye scores**			
Normal	Small amount of ocular discharge	Moderate amount of bilateral discharge	Heavy ocular discharge
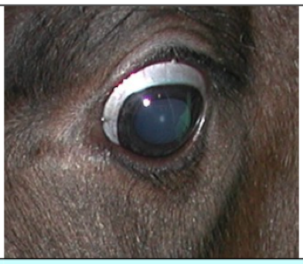	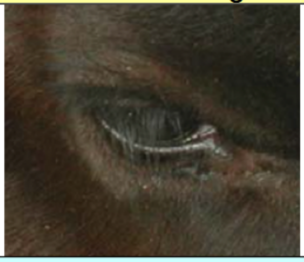	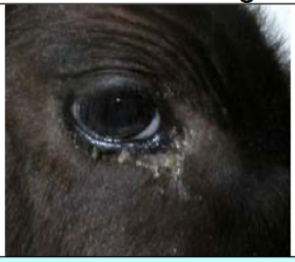	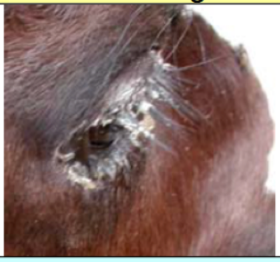
**Ear scores**			
Normal	Ear flick or head shake	Slight unilateral droop	Head tilt or bilateral droop
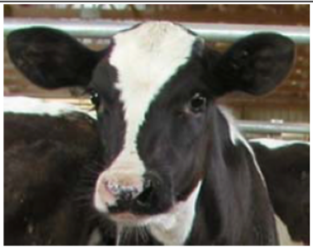	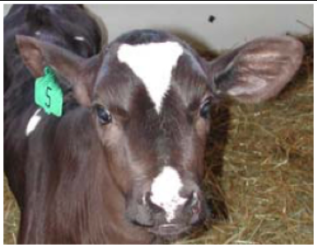	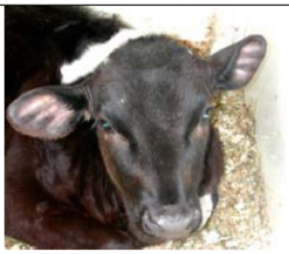	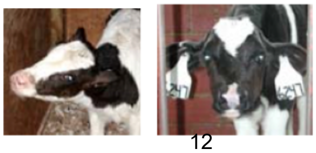

BODY CONDITION SCORE

1-5 for calves/kids/ lambs

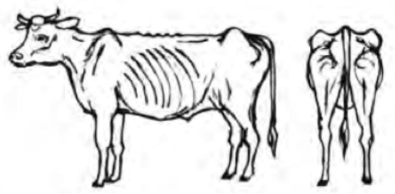

Condition score 1

Backbone prominent
Hips and shoulder bones prominent
Ribs clearly visible
Tail-head area recessed
Skeletal body outline

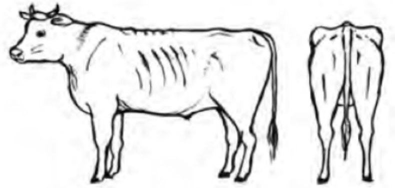

Condition score 2

Backbone visible
Hips and shoulder bones visible
Ribs visible faintly
Tail-head area slightly recessed
Body outline bony

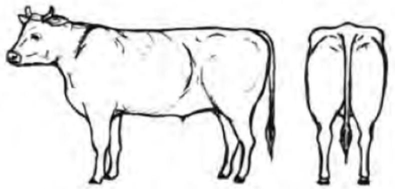

Condition score 3

Hip bones visible faintly
Ribs generally not visible
Tail-head area not recessed
Body outline almost smooth

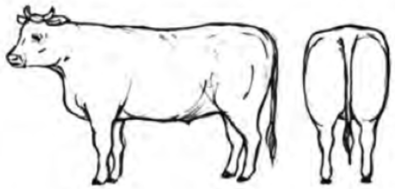

Condition score 4

Hip bones not visible
Ribs well covered
Tail-head area slightly lumpy
Body outline rounded

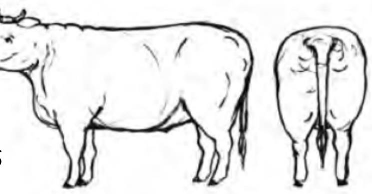

Condition score 5

Hip bones showing fat deposit
Ribs very well covered
Tail-head area very lumpy
Body outline bulging due to fat

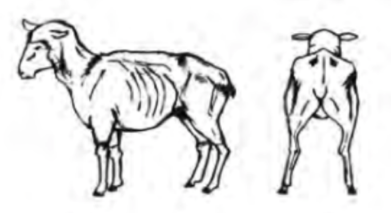

Condition score 1

Appearance angular and narrow
Backbone raised and sharp
Hollow behind ribs
Tail feels bony
Neck bones prominent

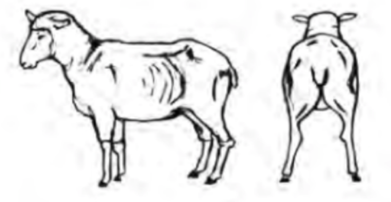

Condition score 2

Backbone raised but smooth
Ribs are easily felt
Tail bone easily detectable
Thin neck

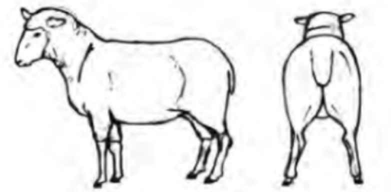

Condition score 3

Backbone slightly raised
Ribs smooth, can just be felt
Tail bones barely detectable

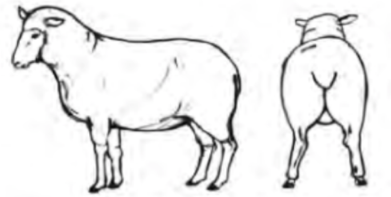

Condition score 4

Appearance well rounded
Backbone can just be felt
Ribs are covered
Tail firm and rounded

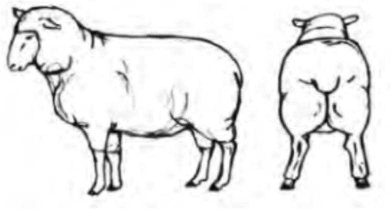

Condition score 5

Appearance very well rounded
Backbone barely detectable
Ribs cannot be felt
Tail fat and broad

https://www.daf.qld.gov.au/__data/assets/pdf_file/0015/53520/Animal-HD-Investigation-Condition-scores.pdf

BODY CONDITION SCORE

0-5 for camel calves

https://doi.org/10.9755/ejfa.v12i1.5193

Emir. J. Agric. Sci. 2001. (13) : 01-06
http://www.cfs.uaeu.ac.ae/research/ejas.html

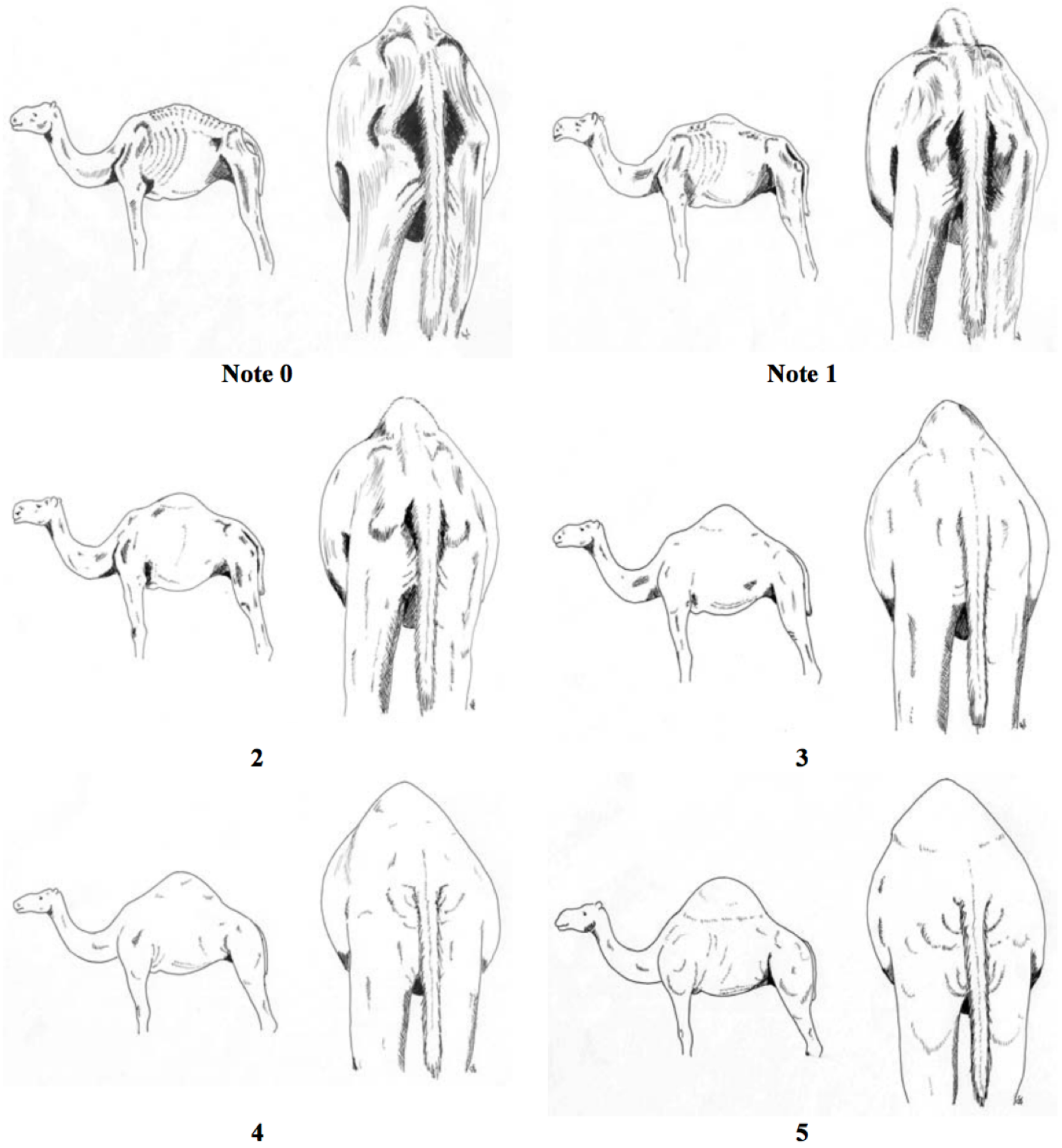

Figure 3. Body condition score (0 to 5) of the dromedary camel

FECAL SCORE

0	1	2	3
NORMAL	Pasty, normal color	Loose	Watery

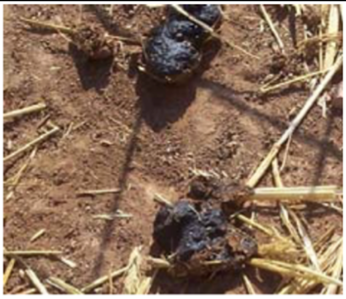
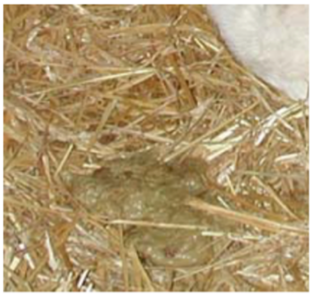
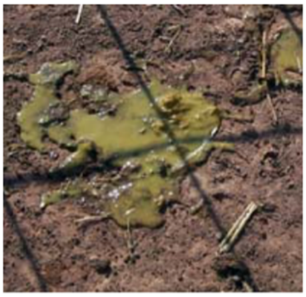
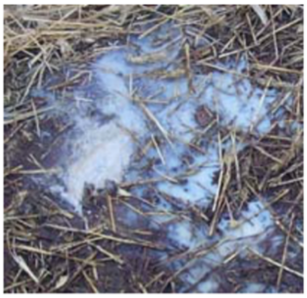

https://fyi.extension.wisc.edu/heifermgmt/files/2015/02/calf_health_scoring_chart.pdf

Intervention arm

Mixed

Questionnaire ID 59 / |__________|

Enumerator |__________|

Date |__________|

Respondent's Data

Surname |__________| Name |__________|

District
Village
Kebele
Address |__________|

Mobile phone |__________|

GPS coordinates
(farm Lat-Long) |__________|

☐ The interviewee understands and agrees with the privacy policy
☐ The interviewee agree to taking part in this questionnaire

Mixed: Calves

50.-Number of adult cows above 2 years of age in the household

|_____________| cows

51.-Number of calves born (both dead and alive) during the last 12 months

|_____________| calves

52.-Number of calves born alive during last 12 months that died

|_____________| calves

53.-Number of calves born dead (stillbirths) during the last 12 months

|_____________| calves

54.-Number of calves born during the last 12 months that died within the first week of birth

|_____________| calves

55.-Number of calves born during the last 12 months which developed navel infection

|_____________| calves

56.-Did you dip the navel of newborn calves with iodine immediately after birth?

O Yes
O No

☐ Multiple choice ◯ Unique choice |__________| Number

57.-Number of calves born during last 12 months that were treated for navel infection

|_____________| calves

58.-Did the calves born during the last year got colostrum in the first day of life?

O Yes
O No

59.-What is the amount of milk fed daily to newborn calves?

O < 1 litre
O 1 -2 litres
O >2 litres

60.-Do you provide milk replacers to newborn calves?

O Yes
O No

61.-Do you provide supplementary feed (other than milk or milk replacement) to non-weaned calves?

O Almost always
O Sometimes
O Always never

62.-When do you introduce supplementary feed different from milk/milk replacer to calves?

O < 1 month
O 1-2 month
O > 2 month
O I don't use supplementary feed

63.-How often do you provide water to non-weaned calves?

O Twice per day
O Oncer per day
O Every other day
O Do not provide water

64.-How many cows had been pregnant during the last 12 months?

|_____________| cows

65.-How many cows had difficult birth (dystocia) in the last 12 months?

|_____________| cows

66.-How many cows in your herd abort in the last 12 months?

|_____________| cows

67.-Of the cows that gave birth in the last 12 months, how many had mastitis or closed teats (no colostrum or milk)?

|_____________| cows

68.-Do you keep pregnant cows separated during parturition?

O Almost always
O Sometimes
O Always never

69.-Do you provide feed supplements for pregnant cows near to parturition?

O Almost always
O Sometimes
O Always never

70.-Are sick calves examined for disease by health personnel?

O Almost always
O Sometimes
O Always never

71.-How many calves develop diarrhea during the last 12 months?

|_____________| calves with diarrhea

72.-How many calves develop respiratory disease during the last 12 months?

|_____________| calves with respiratory disease

73.-Do you have separate calf pens?

O Yes
O No

Mixed: Lambs and kids

74.-Number of adult female sheep and female goats (above 1 year of age) in the household

|_____________| females

75.-Number of lambs and kids born (both dead and alive) during the last 12 months

|_____________| lambs/kids

76.-Number of lambs and kids born alive during last 12 months that died

|_____________| lambs/kids

77.-Number of lambs and kids born dead (stillbirths) during the last 12 months

|_____________| lambs/kids

78.-Number of kids / lambs born during the last 12 months which developed navel infection

|_____________| lambs/kids

79.-Are sick lambs/kids examined for disease by health personnel?

O Almost always
O Sometimes
O Always never

80.-How many lambs/kids develop diarrhea during the last 12 months?

|_____________| lambs/kids with diarrhea

81.-How many lambs/ kids develop respiratory disease during the last 12 months?

|_____________| lambs/ kids with respiratory disease

Pastoral
Questionnaire ID 60 / ____________

Enumerator ____________

Date ____________

Respondent's Data

Surname ____________ Name ____________

District
Village
Kebele
Address ____________

Mobile phone ____________

GPS coordinates
(farm Lat-Long) ____________

☐ The interviewee understands and agrees with the privacy policy
☐ The interviewee agree to taking part in this questionnaire

Pastoral: Calves

100.-Number of adult cows above 2 years of age in the household

|_____________| cows

101.-Number of calves born (both dead and alive) during the last 12 months?

|_____________| calves

102.-Number of calves born alive during the last 12 months that died

|_____________| calves

103.-Number of calves born during the last 12 months which developed navel infection

|_____________| calves

104.-Number of calves born during last 12 months that were treated for navel infection

|_____________| calves

105.-Number of calves born dead (stillbirth) during the last 12 months

|_____________| calves

106.-Number of calves born during the last 12 months that died within 1 week of birth

|_____________| calves

☐ Multiple choice ○ Unique choice ▭ Number

107.-Did the calves born during the last 12 months got colostrum in the first day of life?

O All of them
O Most did
O Many didn't

108.-Number of calves born during last 12 months that died due to malnutrition/feed shortage

|____________| calves

109.-What is the amount of milk fed daily to newborn calves?

O < 1 litre
O 1 -2 litres
O >2 litres

110.-Do you provide milk replacers to newborn calves?

O Yes
O No

111.-Do you provide supplementary feed (other than milk or milk replacement) to non-weaned calves?

O Almost always
O Sometimes
O Always never

112.-When do you introduce feed different from milk/milk replacer to calves?

O < 1 month
O 1-2 month
O > 2 month
O I don't use supplementary feed

113.-How often do you provide water to non-weaned calves?

O Twice per day
O Oncer per day
O Every other day
O Do not provide water

114.-How many cows had been pregnant in the previous 12 months?

|____________| cows

115.-How many cows had difficult birth (dystocia) in the last 12 months?

|____________| cows

116.-How many cows in your herd abort in the last 12 months?

|____________| cows

117.-Of the cows that gave birth in the last 12 months, how many had mastitis or closed teats (no colostrum or milk)?

|____________| cows

118.-Do you keep pregnant cows separated during parturition?

O Almost always
O Sometimes
O Always never

119.-Do you provide supplements to cows approaching parturition?

O Almost always
O Sometimes
O Always never

120.-Are sick calves examined for disease by health personnel?

O Yes
O No

121.-How many calves develop diarrhea during the last 12 months?

|_____________| calves with diarrhea

122.-Number of calves with respiratory problems in the last 12 months

|_____________| calves with respiratory problems

Pastoral: Lambs and kids

123.-Number of adult female sheep and female goats (above 1 year of age) in the household

|_____________| females

124.-Number of lambs and kids born (dead and alive) during the last 12 months

|_____________| lambs/kids

125.-Number of lambs and kids born alive during the last 12 months that died

|_____________| lambs/kids

126.-Number of newborn kids / lambs born during the last 12 months which developed navel infection

|_____________| lambs/kids

127.-Number of kids / lambs born during the last 12 months that died in the first week of age

|_____________| lambs/kids

128.-Are sick kids/lambs examined for disease by a health professional?

O Almost always
O Sometimes
O Always never

129.-How many lambs/kids develop diarrhea during the last 12 months?

|_____________| lambs/kids with diarrhea

130.-How many lambs/ kids develop respiratory disease during the last 12 months?

|_____________| lambs/ kids with respiratory disease

Pastoral: Camels

131.-Number of adult female camels (above 4 years of age) in the HH

|____________| females

132.-Number of camel calves born (dead and alive) during the last 12 months

|____________| calves

133.-Number of camel calves born alive during last 12 months that died

|____________| calves

134.-Number of camel calves born during the last 12 months which developed navel infection

|____________| calves

135.-Number of camel calves born during last 12 months that were treated for navel infection

|____________| calves

136.-Number of camel calves born dead (still birth) during the last 12 months

|____________| calves

137.-Number of camel calves born during the last 12 months that died within one week of birth

|____________| calves

138.-Did the camel calves born during the last 12 months got colostrum in the first day of life?

O All of them
O Most did
O Many didn't

139.-Number of camel calves born during the last 12 months that died due to malnutrition/feed shortage

|____________| calves

140.-What is the amount of milk fed daily to newborn camels?

O Leave one quarter
O Leave half quarter
O Residual suckling

141.-Do you provide supplementary feed (other than milk or milk replacement) to non-weaned camel calves?

O Almost always
O Sometimes
O Always never

142.-When do you introduce feed different from milk/milk replacer to camel calves?

O < 1 month
O 1-2 month
O > 2 month
O I don't use supplementary feed

143.-How often do you provide water to non-weaned camel calves?

O Twice per day
O Oncer per day
O Every other day
O Do not provide water

144.-How many she camels have been pregnant in the last 12 months

|____________| cows

145.-How many she camels had difficult birth (dystocia) in the last 12 months

|____________| cows

146.-How many she camels in your herd abort in the last 12 months?

|____________| cows

147.-Of the camels that gave birth in the previous 12 months, how many had mastitis or closed teats (no colostrum or milk)?

|____________| cows

148.-Do you keep pregnant camels separated when approaching parturition?

O Almost always
O Sometimes
O Always never

149.-Do you provide supplements to pregnant camels when approaching parturition?

O Almost always
O Sometimes
O Always never

150.-Did sick camel calves were examined for a disease by a health personnel?

O Almost always
O Sometimes
O Always never

151.-How many camel calves develop diarrhea during the previous 12 months?

|____________| calves with diarrhea

152.-Number of camel calves with respiratory problems in the last 12 months?

|____________| calves with respiratory problems

Periurban
Questionnaire ID 57 / |____________|

Enumerator |____________|

Date |____________|

Respondent's Data

Surname |____________| Name |____________|

District
Village
Kebele
Address |____________|

Mobile phone |____________|

GPS coordinates
(farm Lat-Long) |____________|

☐ The interviewee understands and agrees with the privacy policy
☐ The interviewee agree to taking part in this questionnaire

Periurban: Calves

1.-Number of adult cows above 2 years of age in the household

|____________| cows

2.-Number of total calves born (both dead and alive) during the last 12 months

|____________| calves

3.-Number of calves born alive during the last 12 months that died

|____________| calves

4.-Number of calves born during the last 12 months which developed navel infection

|____________| calves

5.-Did you dip the navel of newborn calves with iodine immediately after birth?

O Yes
O No

6.-Number of calves born during the last 12 months that were treated for navel infection

|____________| calves

7.-Number of calves born dead (stillbirths) during the last 12 months

|____________| calves

☐ Multiple choice ◯ Unique choice ▭ Number

8.-Number of calves born during the last year that died within the first week of birth

|____________| calves

9.-Did the calves born during the last year got colostrum in the first day of life?

O Yes
O No

10.-What is the amount of milk fed daily to newborn calves?

O < 1 litre
O 1 -2 litres
O >2 litres

11.-Do you provide milk replacers to newborn calves?

O Yes
O No

12.-Do you provide supplementary feed (other than milk or milk replacement) to non-weaned calves?

O Almost always
O Sometimes
O Always never

13.-When do you introduce supplementary feed different from milk/milk replacer to calves?

O < 1 month
O 1-2 month
O > 2 month
O I don't use supplementary feed

14.-How often do you provide water to non-weaned calves?

O Twice per day
O Oncer per day
O Every other day
O Do not provide water

15.-How many cows had been pregnant during the last 12 months?

|____________| cows

16.-How many cows had difficult birth (dystocia) in the last 12 months?

|____________| cows

17.-How many cows in your herd abort in the last 12 months?

|____________| cows

18.-Of the cows that gave birth in the last 12 months, how many had mastitis or closed teats (no colostrum or milk)?

|____________| cows

19.-Do you keep pregnant cows separated during parturition?

O Almost always
O Sometimes
O Always never

20.-Do you provide feed supplements for pregnant cows near to parturition?

O Almost always
O Sometimes
O Always never

21.-Are sick calves examined for disease by health personnel?

O Almost always
O Sometimes
O Almost never

22.-How many calves developed diarrhea during the last 12 months?

|____________| calves with diarrhea

23.-Number of calves with respiratory problems in the last 12 months

|____________| calves with respiratory problems

24.-Do you have separate calf pens?

O Yes
O No

NATIONAL YOUNG STOCK MORTALITY REDUCTION PACKAGE

Standard Operation Procedure (SOP) for mixed production system

MAY 30, 2018

Addis Ababa, Ethiopia

1

VI. CHAPTER IV: CALF MORTALITY REDUCTION PACKAGE FOR MIXED CROP-LIVESTOCK PRODUCTION SYSTEM

Introduction

Calf loss is one the major constraints in the development of cattle for dairy and beef production. The annual calf mortality in the range of 11.9-20.9% reported in mixed crop-livestock production system of Ethiopia cannot be tolerated and needs immediate intervention. High mortality of calves occur during the first 3 months of life mainly due to poor colostrums and milk feeding, starter feeding and housing practices that predispose newborns to diseases mainly causing diarrhea and pneumonia. Developing package and introducing improved calf management practices to farming households would gradually reduce calf losses to an acceptable level and raise the availability of replacement stock.

The aim of the calf mortality reduction package is to address husbandry and health related problems associated with calf morbidity and mortality by implementing improved calf management practices at smallholder household level in the mixed crop livestock production system.

4.1 MANAGEMENT AND HUSBANDRY RELATED INTERVENTIONS

INTERVENTION 1: IMPROVING FARM CLEANLINESS/HYGIENE

Description of the intervention: This intervention is to improve the farm hygiene and minimize the possibility of young calf contamination by disease causing agents by constructing appropriate animal houses or barns with floor design for good drainage; by preparing bedding for young calves from locally available materials such as hay, sawdust, sand/floor, etc. by frequent cleaning of the floor and occasionally disinfecting

the animal house. Farm biosecurity certification system will be developed to be practiced by the extension agents. Experts at woreda level will monitor and assure the implementation of activities.

Skill and knowledge capacity building: The following capacity building activities are proposed:

- Training on house and floor designs and farm biosecurity and safety methods for extension agents. This training should be a skill training.
- The extension agents are expected to provide the above skill training for smallholder farmers
- A separate farm disinfection and precautionary measures during disinfection (chemical handling, environmental concerns, …) will be provided for both extension farmers.

Inputs and delivery mechanisms:

- Local construction materials, detergent and protective wear (apron and boots) will be required for farm hygiene and they can be made available by the producer.
- Disinfectants need to be made available by the public and private animal health posts or veterinary clinics.
- A special budgetary support could be provided by the project to introduce the interventions so that these farms could be used as demonstration sites for further up scaling of the interventions

Action 1: Calf pen and floor design

Note:

- Note that unless a biosecurity measure (which includes appropriate calf pen and floor designs) feasible under the smallholder system is developed and enforced, sustainability of the intervention may be questionable
- Note that this intervention is a preventive measures and preventive measures are by far cheaper than the cost of treatments

Do the following:

- Identify through farm visits the status of cattle barns and calf housing situation
- Decide on the farms that need to construct new calf pens and those that need only to modify
- Advice and technically support farmers to construct appropriate house for calves
- The house need to be with slanted floor (3-5 degree slope) suitable for proper drainage.
- Cemented or paved floor with adequate bedding is appropriate for calves
- ☒ Avoid rough floor and crevices on paved or soil floors that prevent draining of urine and manure

Action 2: Floor disinfection

Note:

- Note that cleaning barns alone may not get rid of disease causing agents
- Occasional Disinfection is mandatory
- The intervention is likely to be adopted as it is not too costly for smallholders

Do the following:

- Clean the floor of the house or barn at least once a day using locally available broom
- Drain and wash the floor with water (if paved) at least once a day
- Avoid accumulation of slurry for longer than 2 days as it serves as medium for development of disease causing microbes.
- Thoroughly spray with commercially available and environmentally safe disinfectant such as bleach at least once in three months

Attention: Read instructions of the manufacturer or seek advice from animal health professional for dilution, application and precaution of disinfectants to be used. Don't directly spray disinfectants on animals, feeds, water, feeding and watering troughs to avoid toxicity.

Pre-requisites:

- The required inputs (especially disinfectants) need to be available for the interventions to be successfully introduced
- Continued follow-up and awareness creation is required for interventions to be adopted as farmers' regular practices
- Farmer rewarding mechanism will be developed for best performance through certification.
- A monitoring and evaluation team need to be organized at kebele and district level.

- Skill training and awareness creation on controlled breeding and estrus synchronization to extension agents and farmers is required.
- Training on heat detection will also be given to farmers.

Inputs and delivery mechanisms: Input for estrus synchronization (eg hormones, pregnancy diagnosis kit), burdizzo are to be provided by the public livestock development office.

INTERVENTION 2: REPRODUCTIVE MANAGEMENT

Description of the intervention: The purpose of this intervention is to arrange the mating and calving season to match with forage availability and low disease burden to maximize calf survival.

Skill and knowledge capacity building:

- Skill training and awareness creation on controlled breeding and estrus synchronization to extension agents and farmers is required.
- Training on heat detection will also be given to farmers.

Inputs and delivery mechanisms: Input for estrus synchronization (eg hormones, pregnancy diagnosis kit), burdizzo are to be provided by the public livestock development office.

Action 1: Control of mating/calving

Note:

- Controlled mating and calving by selecting appropriate seasons for maximum calf survival could be achieved:
- By restraining bulls from mating during unwanted seasons, or
- By synchronizing the oestrus period of village cows using hormonal treatment
- It should also be noted that selection of appropriate bulls matching the heifer/cow size would help avoid oversized fetuses that lead to dystocia.

Do the following:

- Identify the most appropriate season for maximum calf survival in consultation with farmers and based on research results if studies relevant for the locality or similar agroecology are available
- Identify the breeding male or use artificial insemination (AI)
- Castrate bulls unwanted for breeding starting from 2 years of age
- Separate breeding male/ young bulls outside of the identified breeding season
- Introduce and strictly follow estrus synchronization protocol of the MoAL for cows/heifers
- use teaser bulls for heat detection
- Avoid breeding malpractices such as opening the cervix with stick/ any foreign materials or follow septic procedure.

Pre-requisites:

- Strictly following estrus synchronization protocols is required for successful implementation of the intervention
- Support from Research centers and universities required to provide training on estrus synchronization and conduct research to refine estrus synchronization protocols

- Regular monitoring and evaluation system is required as described for the other interventions.

INTERVENTION 3: PRENATAL CARE OF THE DAM

Description of the intervention: Healthy calves start with properly conditioned cows that are fed a balanced diet. Cows in proper condition will get off to a better start and will be better able to nourish their calves throughout the season. The prenatal intervention is to give the necessary care for the pregnant cow during the last trimester and at parturition. Interventions include proper feeding of dam during the last trimester (using urea treated crop residues, multi-nutrient blocks, urea-molasses blocks where possible to support the energy, protein, mineral and vitamin A requirement of the dam); retaining the dams around the homestead one week before pending parturition, and one week after parturition, providing a separate clean and dry barn/house for calving, assist during parturition and manage dystocia cases.

Skill and knowledge capacity building: skill focused training shall be delivered for animal extension agents and farmers on preparation of Urea-Molasses blocks, Multi-nutrient blocks, Urea treated crop residues, and production, conservation and use of improved for ages that can be used to feed the dam approaching parturition.

Inputs and delivery mechanisms: Materials needed to produce the various feed supplements. Urea-Molasses lick and urea treated residue can be introduced as a business initiative (with credit availability, tax relief, provi sion of land for the business etc). Alternatively, potential cooperative initiatives can also be applied for production at reduced cost. Production of targeted marketable mineral licks (Limestone, local salt licks) can also be another option. Farmer training and awareness creation is required in areas unlikely to easily adopt the technology such as use of urea treated straw, mineral licks, etc.

Pre-requisites: Accessibility of molasses, regular monitoring system for implementation and sustainability (EAs will be primarily responsible for monitoring the implementation and should be exempt from other Extension work). Intervention enforcement mechanism must be set in place to achieve maximum success

Action 1: Feeding the dam a balanced diet during the last trimester (after six months of pregnancy)

Note:

- ☞ Management decisions made during the last trimester of pregnancy are critical and can influence success at calving, survival of the newborn and profitability of the farm.
- ☞ Underfeeding cows prior to calving could increase calf scours and mortality and reduce calf survival
- ☞ Ideally, most cows should enter the calving season with a body condition score (BCS) of 5 (on a 1 to 9 scale??). If the cows are thinner than desired, it is advisable to alter the plane of nutrition to add body condition prior to calving.

Do the following:

The following points are worth noting during prenatal feed management of the dam

- Feed the dam with fortified (Cu, Mg) Urea-Molasses lick during morning and at night with ample amount of water supply***(Refere to ESGPIP technical bulletin No.1)***, **or**
- Feed legume supplement (Alfalfa, Sesbania, leucaena, etc.) daily by mixing one portion of legume to two parts of dry roughage/grazing, **or**
- Feed a daily supplement of about 1-1.5kg concentrate toeach pregnant dam, **or**
- Feed locally available feeds such as "*Atela"/*areki(from local beer,beverage production) with straw, **or**
- Feed the dam urea treated straw ***(be aware that a straw-only diet can reduce colostrum quality in the cow). (Refere to ESGPIP technical bulletin No.2)***

- Never feed crushed or water dissolved mineral lick to avoid the risk of over consumption by the animal.

Attention:

- **Use reference manual for urea treatment of crop residues, preparation of Urea-Molasses Blocks and production of improved forage.**
- **Make sure your cows or heifers are neither too fat nor too thin at calving**
- **Don't feed Urea-Molasses licks to young calves less than 6 months of age**
- **Do not keep the lick in a wet/moist place**

Action 2: Retaining the dams around homestead

Note:

- Minimizing stress around the time of stress helps both the dam and the new born
- Long distance trekking in search of water and grazing during he last few weeks of parturition is one of the stressful condition

Do the followings:

- Retain the pregnant dam at least one week before expected parturition date and another one week after parturition for proper management of the dam during and after delivery and for maximum care of the newborn calf.
- Keep pregnant cows approaching parturition around the homestead with appropriate feed
- Never allow to travel long distances with the rest of the herd for grazing/watering
- Provide a separate clean and dry pen/house for the pregnant dam during parturition
- Provide plenty of fresh clean water immediately after delivery, supplement with moderate amount of good quality roughage for the first two days and gradually introduce 1kg of concentrate per cow/ day for the remaining days and reduce the concentrate gradually.

- Continue the lactation supplementation to the dam during the pre-weaning period

Action 3: Care of dam and calf at parturition

- Move pregnant animal to the calving unit before calving begins for it reduces stress during calving
- Inspect the animal approaching calving at least twice daily
- Estimate time of parturition by observing signs for approaching (relaxation of pin bone ligaments at the base of tail, full udder and serous discharges from the vulva) (picture, banner)
- Check for labour, restless -lying and standing frequently
- Check for rupture of membrane/water bag, normal presentation of front legs and head of calf
- Give more time to calve (if calf normally presented and cow straining intermittently)
- Intervene if the calf is not born within 1 hour after the water bag or fetal hooves have appeared. Assist after disinfecting hands (e.g. Calf with head deviated or leg down).

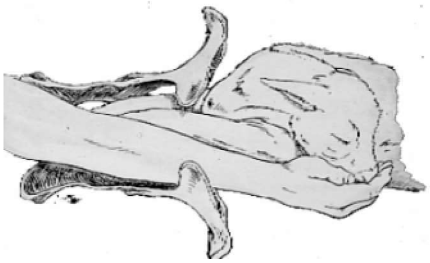

Correction of deviated head by grasping the muzzle or nose of the calf

- If there is abnormal presentation (calf coming backward, oversized calf, died or deformed calf, smelly or bloody discharge) seek help of the local veterinarian. (Preparation of a guideline for dystocia handling, Phantom demonstration of procedure)

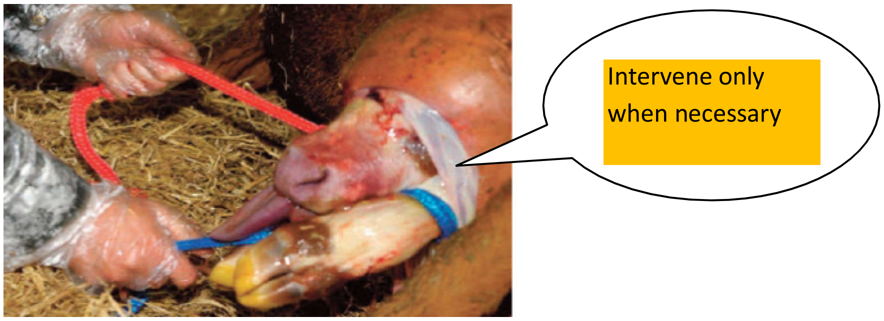

- **Ensure the cow is standing before attempting to correct a wrongly presented calf**
- **Put ropes on or above the fetlock, ensuring that the knots are not on the sides of the legs.**
- **Only pull when the cow forces and relax the ropes when she relaxes**
- **Pull on alternative legs until the head emerges and rotate the calf after the chest emerges to prevent a hip-lock.**

Pre-requisites: preparing calving pen/ house, detergents, antiseptics, apron and gloves, obstetrical kit are required, regular monitoring of implementation by the extension agent, encourage farmers by putting a reward mechanism in place.

INTERVENTION 4: NEONATAL CARE

Description of the intervention: Optimal neonatal care is very important to reduce early mortality because drastic environmental change for the young calf from womb to external environment is one of the major causes of early mortality (in the first 72 hours) especially during cold seasons for weak and premature calves. This neonatal care is critical in the first month of a calf's life as most of calf deaths occur during this stage of life. Management of a calf at birth greatly affects lifetime performance. Protecting the calf’s immune system is of utmost importance. Activities include induction of young calf breathing through clearing the air ways and chest massaging, navel treatment, improving peripheral circulation, feeding colostrum and measuring birth weight.

Note:

- Most of calf deaths occur during the neonatal period
- Management of a calf at birth also greatly affects lifetime performance
- Optimal neonatal care is important to reduce early mortality and protect the calf's immune system
- Drastic environmental change for the young calf from womb to external environment is one of the major causes of early mortality (in the first 72 hours)
- This is especially important during cold seasons for weak and premature calves..

Skill and knowledge capacity building: training will be delivered to extension agents and farmers on appropriate handling of the newborn calf; how to induce calf breathing, adequate and timely colostrum feeding and preparation of replacer colostrum, warming or brooding of calves especially under cold weather conditions.

Inputs and delivery mechanisms: Plastic bulb pipetters used for removing fluid from the nostril and oral cavity of the newborn calf, brooder box with bedding, warm cloth, nipple milk feeder, foster colostrum or colostrum replacers in case of dead dam, detergents, antiseptics, clean thread and blades for navel care, w eighing scale, protective wears for professionals.

Pre-requisites: Accessibility of supplementary feeds, Regular monitoring system for implementation sustainability (EAs will be primarily responsible for monitoring the implementation and those assigned to this work are expected to be exempted from other assignments to concentrate on this work.)

Action 1: Induction of breathing in the newly born calf

Note:

- Survival of the newborn is dependent on the rapid onset of normal, spontaneous respiration.

- In a normal calf if parturition occurs normally, respiration usually commences after 30 seconds of birth, being irregular at first before settling down to 45–60 breaths per minute.
- Once birth is complete, it is important first to ensure that the upper respiratory tract is cleared of fluid, mucus and attached fetal membrane. This can be done with the aid of fingers or, preferably, with a simple suction device (plastic bulb pipette)

Do the following:

- ✓ Start assisting the dam and the calf after washing your hand or use protective gloves at all times
- ✓ Clean the nostrils from any mucus or fluid by gently squeezing the muzzle
- ✓ Hold the newborn by the hind legs and lift upside down for a few seconds to expel fluid from the lungs (as shown in Fig. 4)
- ✓ Insert a finger into the mouth, or gently pull out the tongue until the newborn produces sound
- ✓ Brisk rubbing or gentle massage of the chest with towels frequently provides the necessary tactile stimulus to stimulate respiration.
- ✓ Provide extra care for premature, weak and small calves or seek help of an animal health professional
- ☒ Don't insert sharp and dirty material into the nostril to induce sneezing as this may cause injury

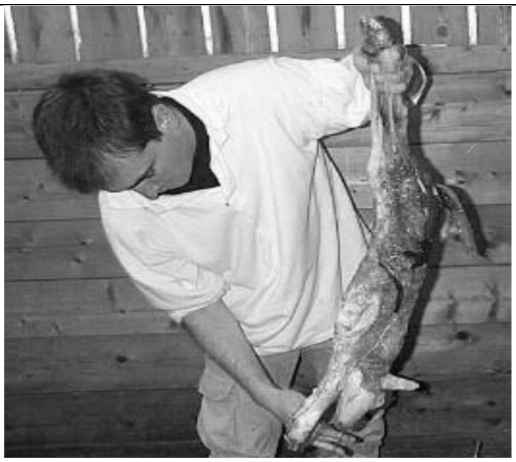

Holding the calf by the rear and removing the mucus from the nostril

Action 2: Preventing navel ill

Note: The umbilicus usually ruptures passively in cattle. It is hence important to handle it properly.

Do the following:

- Tie the umbilical cord at 5 cm (approximately three fingers) length with clean string or thread (Fig. 5a)
- Cut the remaining part of the umbilical cord and dip or rub the stump with antiseptics (iodine tincture or savlon) (Fig. 5b) or apply antibiotic spray (Fig. 5c)
- Carefully observe the umbilicus to dry and heal over the next few days. If it has not dried and sealed off, immediately seek the help of an animal health professional
- Ensure the calf is born in a clean, freshly bedded calving unit
- Tie the umbilical cord at 5 cm (~three fingers) length from the belly with clean string or thread to minimize bleeding and damage to the umbilicus.
- Cut the remaining part of the umbilical cord and dip the stump into available antiseptics (preferably iodine tincture or savlon) or apply wound spray.

- Never paste the umbilicus of the fetus with cow dung for it causes contamination of the navel causing navel ill.
- Check regularly for navel ill in the first week; check for excess bleeding, pain, abnormal swelling, odor or pus
- Seek help of the local animal health professional for treatment if swelling or abscess develops. Treat as recommended by the professional.

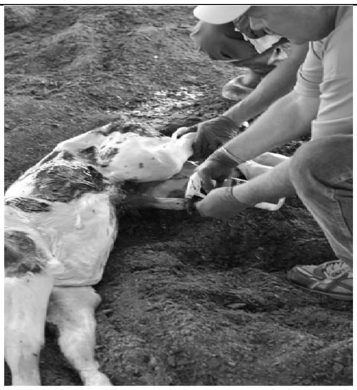 Tie the umbilical cord	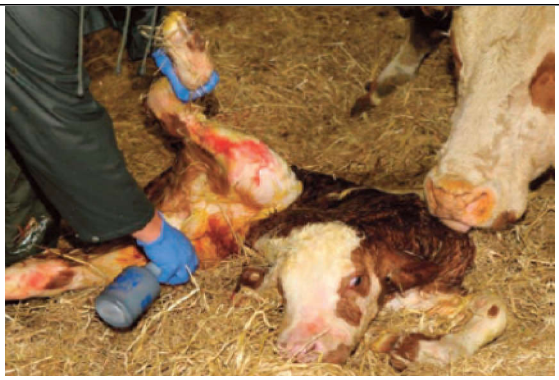 Dip in the navel stump with iodine tincture

Action 3: Improving thermoregulation

Note:

- ☞ Hypothermia (abnormally reduced body temperature) occurs when the energy metabolism is insufficient to maintain body temperature. Following birth, the body temperature of the newborn falls quickly.
- ☞ The newborn has little subcutaneous fat and hence insulation is poor.
- ☞ The body surface is wet and thus heat is lost due to evaporation.
- ☞ It is, therefore, very important to stimulate the calf's peripheral circulation to prevent hypothermia. Hypothermia can be reduced in two ways, by improving feed intake for energy production and by reducing heat loss through insulation.

Do the following:

- Ensure that there is adequate milk intake

- Dry the entire body of the newborn with a clean cloth or allow the dam to lick the calf
- Move the newborn andits dam to a dry and clean place with good bedding

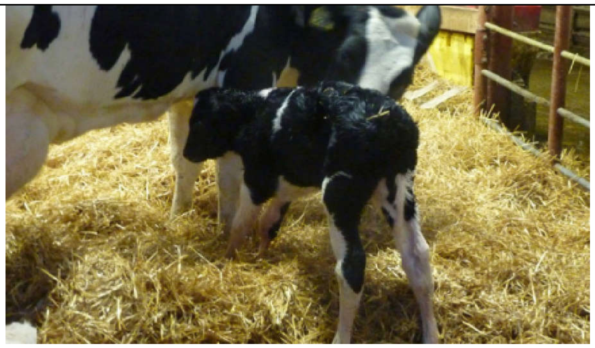

Keeping calf with dam in clean place with good bedding made from straw

- If the ambient temperature is cold or if the newborn is too weak to stand, provide warmth (with coal fire or wrap with piece of blanket or old woolen cloth or gunny bag)
- Avoid overheating of the newborn or don’t continuously heat for toolong. Observe the comfort of the calf.
- Never pour cold water on the body of the newborn calf as moisture can aggravate hypothermia.

Article 4: Colostrum feeding (1-4 days)

Note:

- ☞ Colostrum is critical to survival of the newborn calf.
- ☞ The immune system of newborn calves is not completely developed and the immunoglobulins in colostrum contribute a substantial component of the immune protection in newborn calves.

Do the following:

- Check if the dam is producing sufficient colostrum by test-milking all the teats

- Make sure the newborn suckles the dam immediately or give colostrum within two hours after calving, within 3-6 hoursat the latest.
- Give at least 2 liters of colostrum on the first feeding
- Make sure free suckling for at least 4 days (colostrum should be considered as the first vaccination of the calf)
- If the dam does not have enough colostrum supply foster colostrum from a contemporary cow, or artificial colostrum (***preparation:1 egg beaten+500ml fresh warm water+500ml of whole milk+1tea spoonful of oil***) as substitute and feed the mixture 3 times a day for 3 days.
- If the newborn is not suckling for any reason (weak and/or premature), milk the dam or foster dam and feed using milk feeding devices such as a nipple feeder, catheter syringe, or a stomach tube.
- About 2-3 liters of colostrums/day/per calf is required depending upon the size of the calf.

TIPS:

- **Use colostrum from the first milking as the first feed**
- **Give colostrum within 2 hours from the calf's birth for local and crossbred calves**
- **Never delay colostrum feeding beyond 6 hours after birth for it is rich in nutrients and antibodies and many diseases could be prevented by early colostrum feeding.**
- **Never use animal colostrum for human consumption to avoid human competition)**
- **Don't separate the calf from the dam in the first 4 days of life**

Action 5: Measuring/assessing the birth weight, health status and keeping records

Note:

- Measurements taken at birth are very important to make decisions whether the calf requires further assistance.

- Birth weight of a calf is known to affect its survival chance, calves weighing far less than the average birth weight of the breed are highly likely to die during the neonatal period
- It is important that development agents/veterinary assistants be informed of the average lamb/kid birth weights of the breeds in their locality

Do the following:

- Measure body weight with a weighing scale where available
- Subjectively assess the weight/condition category as "Very small", "Normal", or "Large" size where a weighing scale is not available
- Provide extra neonatal care for premature, very small or underweight calves
- Assess the health of the newborn from body reflexes (palpebral reflex: touch the eyelid; anal reflex: touch the anus, or pedal reflex: pinch the interdigital skin) and seek help from animal health professional for sick offspring
- Record all other birth data (date of birth, weight, and condition of birth) on a predesigned format
- ☒ Don't herd or keep weak and small calves with adults other than the dam at any time at least for the first week of life
- ☒ Don't separate calves from the dam at least for the first week of life to strengthen the dam-offspring bond

Pre-requisites: Avail disinfection materials (iodine tincture), regular monitoring of implementation by the extension agent, introduce farmer rewarding mechanism for active participation.

INTERVENTION 5: MILK FEEDING MANAGEMENT OF CALVES

Description of the intervention: this intervention is to provide the necessary nutritional and health care for the young calf during the pre-weaning period from week one to 3 months of age. At this stage of life, the calf should get sufficient milk for its growth. The future performance of the animal depends mostly on the feeding system at

calfhood. Succulent grass and legumes must be ready for calves starting from 3 weeks of age for early development of the rumen. close monitoring of the health status of calves is needed as parasitic diseases and infectious diseases are common at early age . Activities in this intervention include appropriate milk feeding, preferential and separate feeding (creep feeding); deworming, early detection and immediate treatment of sick calves.

Skill and knowledge capacity building: Trainings will be given for extension agents and farmers on construction of creep feeders, method of deworming, early detection of signs of diseases in calves and first hand management or nursing of sick animals.

Inputs and delivery mechanisms: anthelminthics, oral rehydrating solutions, antibiotics will be available in local animal health posts both in public and private.

Action 1: Appropriate milk feeding

Note:

- ✓ Milk contains all the necessary elements to meet the demand by the newborn calf. It is thus essential that the newborn consumes adequate milk during this period.

Do the following:

- Check if the dam is producing sufficient milk by test-milking the teats
- Allow the calf to suckle leaving at least one of the quarters from 5days to 3 weeks of age.
- If the dam does not produce enough milk or is not willing to nurse the calf, or dies , implement foster milk feeding
- If the newborn is not suckling for any reason, milk the dam and feed using milk feeding devices such as nipple feeders, catheter syringes, or stomach tubes (2-3lts of milk or skim milk is required/calf /day upto 1 month of age.
- Feed the total amount of milk allowance at 3 or 4 equal intervals. The amount of milk can be gradually reduced by feeding increasing quantities of calf starter and lush forage.
- Follow the following feeding guide for calf:

- 4 days-4weeks: 2.5kg milk, 50g calf starter and free choice green grass;
- 4 -6 weeks: 3.0kg milk, 50-100g calf starter and free choice green grass;
- 6-8 weeks: 2.5kg milk, 100-250g calf starter and free choice green grass;
- 8-10 weeks: 2.0kg, 250-350g calf starter and free choice green grass;
- 10-12 weeks: 1.5kg, 350-500g calf starter and free choice green grass;
- 12-16 weeks: (Weaning) 500-750g calf starter and free choice green grass.
- Give the calf its milk immediately after milking when the milk is at body temperature to avoid digestive troubles.

- Fix a poster containing the information/guideline above on the wall of the farm for easy reference
- Train the calves to feed from buckets and start eating calf starters
- To train the calf to consume from a bucket, put two fingers in the calf's mouth so it starts to suck, gently lower its mouth into the colostrum/milk in the bucket, slowly. Control the bucket feeding of milk as greedy calves can swallow large quantities of milk some of which may end up in the windpipe leading to pneumonia.
- To train the calf to consume calf starter concentrate, rub a small amount of starter on the calf's mouth, after milk feeding for a few days.
- Feed the calf only milk up to 3 weeks of age
- Place hay, water and protein supplement (such as high quality lush forage) near the calf to encourage early eating and drinking starting from 3 weeks of age. This helps development of the rumen.
- Observe that the composition of starter feed supplements fulfils the following:
  - *Calf starter:* Calf starter is a highly nutritious concentrate mixture formulated to contain all the nutrients in proper proportion required for optimum growth and is used as a partial substitute for whole milk in the ration of calves. The starter should contain 18% crude protein, low fibre, 0.7% calcium, 0.45% phosphorus and vitamins A, D and E as the calf is consuming little milk and its rumen is still not fully developed. Since quality of protein is very important to calves until their rumen is fully

functional, it is good to include animal protein supplements such as fish meal in calf starters. Urea should never be included in calf starters. The following table presents a simple calf starter formula.

Example of a simple, acceptable calf starter.

Ingredient	% of total ration
Corn, cracked	52.0
Oats	20.0
Soybean meal	20.0
Molasses	5.0
Limestone	1.0
Dicalcium phosphate	0.25
Salt, trace mineral	0.20
Animal fat	1.50
Vitamin supplement	0.05 (or to provide needed vitamins)
Other (coccidiostat, buffer)	as needed

- *Roughage:* only high-quality forages supplemented with concentrates (calf starter) early in life should be offered. If hay is used, it should be of high quality, fine texture, mixed with legumes and fed *ad libitum*. If calves are on pasture, it is best to always graze them ahead of adults to minimize parasite infestation. Common roughages offered to calves include sweet potato vines and freshly harvested, wilted legume forages like Alfalfa and similar high quality forages.
- *Water:* Calves should be offered fresh water *ad libitum* in addition to milk. It is estimated that efficient feed conversion requires about 4 kg of water for each kilogram of dry feed the calves eat.

- You can use different calf feeding methods as appropriate. The following are the most common methods:
    - ***Individual suckling:*** The natural way for a calf to feed is to suckle the dam after she is milked. Do not completely milk the cow out or there will be no milk left for the calf. A simple way to feed the calf is to leave one quarter unmilked and allow the calf to suckle this quarter after each milking. Leave the calf with the dam for around two hours and then remove it until after the next milking.
    - ***Foster mother or multiple suckling:*** One milking cow may be assigned a number of calves to suckle, depending on the level of milk production. This is practical only on farms with several cows lactating at the same time.
    - ***Nipple suckling:*** A rubber nipple is fixed on a milk bottle and the calf is trained on how to suckle. The alternative is to put milk into a bucket and insert a flexible plastic hose pipe with one end attached to a nipple.
    - ***Bucket feeding:*** The most commonly used method is bucket feeding. The calf is trained to drink milk from a bucket. Stainless steel buckets, where available, should be used for hygienic reasons as plastic buckets are difficult to clean.
- Wean the calves on high-quality pasture and/or fodder to maintain high growth rate.
    - A concentrate mixture of (e.g. 35% oats, 5% linseed cake, 30% bran, 10% corn, 20% groundnut cake) may be fed to the calves.
    - It is good to hang a mineral lick where the calf can get the minerals it needs.
- Don’t feed milk from clinically mastitic cows
- Don’t feed excess milk to the calf, it can cause scouring. If scouring occurs, reduce the amount of milk by half and mix the remainder with an equal amount of water.

Action 2: Preferential and separate calf feeding

Note:

- ☞ As the calf grows, its nutrient requirement also grows. However, it can be difficult to reach a maintenance feeding level when there are competitors.
- ☞ The calf requires a separate extra feeding care that can be provided as follows

Do the following:

- Use creep feeding starting from 3 weeks of age
- Use only fresh feed daily
- Always use clean milk feeders and other feeding and watering equipment
- Provide concentrate after feeding roughages
- Don't over feed concentrates to young calves
- Don't feed moldy feed to the young calf
- Never use urea treated roughage for calves before 3 months of age

Pre-requisites: Accessibility of supplementary feeds, advising farmers in the preparation of fresh local feeds (roughages and concentrates) appropriate for young calves, regular monitoring of implementation by the extension agent, introduce farmer rewarding mechanism for active participation in good handling of calves.

4.2 HEALTH RELATED INTERVENTIONS

INTERVENTION 1: PREVENTION AND CONTROL OF DISEASES CAUSING DIARRHEA IN CALVES

Description of the intervention: Acute diarrhea accounts for approximately 75% of the mortality of dairy calves under 3 weeks of age.

The main objective of this intervention is to introduce prevention, control and management strategies to reduce calf mortality due to diarrhea. This requires several steps among which vaccination of calves, vaccination of the cow to boost passive

transfer of immunity, Adequate and timely Colostrum feeding, availing isolation and quarantine area, rehydration of scouring calves and seeking help of animal health professionals for treatment of sick calves

Diarrhea is not always the result of infectious agents. It can also be the result of stress, poor management and poor nutrition.

Neonatal diarrhea could be caused by nutritional disorders, viruses or bacteria; digestive upsets leading to scours are generally a major cause of death in young calves.

The most important pathogens associated with calf diarrhea are enterotoxigenic *Escherichia coli*, rotavirus, corona virus, *Cryptosporidia* sp. *Salmonella* sp. and Coccidiosis (*Eimeria* sp.).

- *E. coli* may affect calves as young as 16 to 24 hours of age but it is most common in calves 1-7 days old.
- *Clostridium perfringens* type C is most common in calves 1-3 weeks of age. This bacterium is a soil born organism and causes calf scours on many unhygienic farms.
- Salmonella typhimurium is the most common cause. Calves are usually affected at 1-7 days of age. The source of Salmonella infection in a herd can be from other cattle, birds, cats, rodents, water supply or human carriers.
- Rota virus infection can occur in calves as young as 0-28 days old; however, most outbreaks occur when calves are 1-6 days old
- Coronavirus can occur in calves 0-28 days old but it is most common in calves of 7-10 days of age. The virus is not as resistant to degradation as Rotavirus. Adult cows shed the virus in feces and contaminate calving areas. Rota- and coronavirus are hardy and survive for months in moist and cool conditions
- Bovine viral diarrhea (BVD) virus infection is common in young calves. Diarrhea begins about 26 hrs to 3 days after exposure and may persist for quite a long time

- Cryptosporidium is most common in calves 1-3 weeks of age. As a rule, cryptosporidium is detected in combination with coronavirus, rotavirus, and/or E. coli.
- Coccidiosis occur in calve 1 week of age. However; outbreaks associated with stress, poor sanitation, over-crowding, or sudden changes in feed in calves 3-4 weeks of age and older have been reported.

Skill Capacity building: awareness creation, training and demonstration related to prevention of diseases of calves causing diarrhea, management of diarrheic calves, preparation of rehydrating solutions locally will be given to extension agents and farmers.

Inputs and delivery mechanisms: The following inputs are required

- Vaccine: bovine E coli vaccine (K99 bacterin), bovine rotavirus vaccine and bovine corona virus vaccine
- Antiparasites: Albendazol, Tetramizol,, Fenbendazol, Decoquinate, Sulfadimidin, Amprolium, Monenzine sodium
- Antibiotics: Penicillin (also as Penstrep), Neomycine, Spectinomycine, , SMZ-TMP (Bacterium) tablets
- Supportive treatments: Oral rehydration salt (ORS), flunixin meglumine (Benamine), Normal bovine plasma, Dextrose saline, normal saline, ringers lactate
- Antiseptics: Formalin (10%), Virocyl, Bleach, Savalon, Alcohol (denatured), Detergents

Materials/equipment: protective clothing, vaccination and treatment syringe and needle, sprayer, cotton, gauze, gloves

Action 1: Vaccination

Note:

- Vaccination helps the calf to cope with diseases challenges in the new environment.

- Prevention of diarrhea can be made indirectly by vaccinating the pregnant dam or by directly vaccinating the young.
- Vaccinate the dam close to the time of parturition and then timely provide adequate colostrum to the calf to give protection for few months until the calf itself is able to produce antibodies.
- The age at which vaccination is made for the calf may vary for different diseases. Usually colostrum must be fed within few hours of birth while vaccination of calves is made after eight weeks of age.

Do the followings:

- Vaccinate the breeding female before pregnancy to strengthen passive transfer of immunity through the colostrums right after delivery
- Follow the instructions by the manufacturer for vaccinations
- Don't vaccinate pregnant cows/heifers at early stage of pregnancy and calves before six months of age
- Ensure passive immunity through adequate and timely provision of colostrums (***refer to the SOP for colostrum feeding in this manual***)
- Vaccinate all calves above 8 weeks of age for bovine *E coli* vaccine (K99 bacterin), bovine rotavirus vaccine, corona virus, and BVD vaccine
- Vaccinate the pregnant female around one month before parturition with bovine E coli vaccine (K99 bacterin), bovine rotavirus, corona virus vaccine, and BVD vaccine to strengthen passive transfer of immunity through colostrum
- Preferably use 2ml, IM injection (side of the neck) of ***Rotavec® Corona*** vaccine that can protect against Rota, Corona and E coli
- Follow the instruction by the manufacturer for vaccination and withdrawal

- ***Generally, the following steps might apply for vaccination***
  - Read the labels on the vaccine vial every time you are about to vaccinate even if you know the label by heart

- Record in a notebook which calf you are vaccinating, the date and the vaccine batch to avoid mistakes of revaccination
- Use the proper syringe and a needle (18 gauge and at least 2 cm long)
- Disinfect the needle between vaccination if you are not using disposable needles
- Inject the needle on either side of the neck, pinch the skin and inject 2 ml vaccine parallel to the skin (SQ) and repeat as prescribed for the vaccine
- Make sure you store the vaccines properly usually refrigerated at +4 degrees
- Unused vaccine must be discarded. Partially used vaccines will be contaminated and are dangerous for the animal if used
- If you inject yourself mistakenly, seek help of a physician immediately
- Don't inject needle in dirty area otherwise it might become infected
- It is good to replace the vaccination needle once for every 20 animals
- Don't vaccinate pregnant cows/heifers in the first trimester (first 3 months of pregnancy)
- Don't vaccinate unhealthy and wet animals
- Don't wipe injection sites with disinfectant, this can reduce the effectiveness of the vaccine
- Don't give other medication at the time of vaccination

Action 2: Isolation and quarantine

Note:

- Remember some diarrhea causing agents could be shed to the environment even before clinical symptoms are apparent and during convalescence (recovering animals). This can contaminate the environment and hence other susceptible animals.
- Separating the sick calf helps to give sufficient attention for care and prevention of the spread of the diseases.

- Close observation of calves at feeding to identify scouring animals as early as possible for remedial treatment will prevent dehydration and secondary disease leading to chronic ill-thrift and mortality.

Do the followings:

- Avail a separate, dry and clean barn for isolating sick animals
- Newly introduced animals (purchased or returned from market) should also be separated for one week
- Quarantined sick animals must be fed and watered using a separate trough and kept until the illness has gone
- Provide proper care (good feed and water; dry and warm bedding and well ventilated) and seek help of animal health professionals for treatment/management of isolated animals
- Clean (remove the feces with brush/spade and wash with detergent water) and disinfect the barn using bleach or 10% formalin if the floor is contaminated with feces
- If a calf is dead, properly dispose by burying or burning following the recommendation of animal health expert (awareness creation on disposal/training (one disposal pit/village may be required)
- Be sure equipment, boots and hands are thoroughly cleaned after handling sick animals
- Don’t mix sick and apparently healthy animals in the isolation barn
- Don’t delay to seek help of animal health professionals for cases of diarrhea

Action 3: Rehydration of diarrheic calves

Note:

- It is essential to replace lost fluid due to diarrhea as a first measure to save the life of the calf.

- The critical to remember that dehydrated calves need 6.5 to 8 liters of fluid daily to correct the dehydration. They also need an adequate calori c intake for maintenance and growth

Do the following during rehydration of the calf:

- ✓ Provide oral rehydration salts (readymade/home prepared) immediately upon detection of diarrhea (10% of BW three times/day) until diarrhea stops
- ✓ Prepare homemade rehydration solution as follows
  - ✓ **Solution 1**
    - 6 tablespoonfuls of sugar
    - ¼ tablespoonfuls of salt
    - Mix into 1 liter of clean water and use 7 or 8 liters every 24 hours

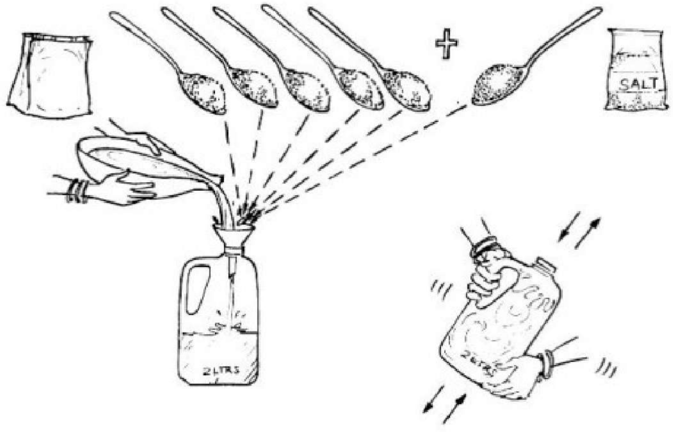

  - ✓ **Solution 2**
    - ¼ tablespoonfuls of salt
    - ¼ tablespoonfuls of baking soda (optional)
    - 6 tablespoonfuls of sugar
    - 2 tablespoonfuls of lemon juice (optional)
    - Mix into 1 liter of clean water and use 7 or 8 liters every 24 hours
- ✓ Rest diarrheic calf until recovery
- ✓ Don't stop provision of ORS before diarrhea stops
- ✓ Oral rehydration salt (ORS) is a supportive treatment hence don't consider ORS as a final and curative treatment

- Don’t give antibiotics to treat scours resulting from over-feeding or digestive upsets. Blood scours (mostly caused by coccidian or other microorganisms) require veterinary treatment and management changes to improve hygiene.

Action 4: Prevention and treatment of sick dams and calves

Note:

- Before treating a scoring calf, it is essential to determine why it is scouring. Take the calf's temperature using a rectal thermometer. If body temperature is above the normal range (38.5-39°C), fever medications such as Flunixin meglumine and antibiotics must be given.

Do the followings:

- ✓ Employ the necessary standard hygienic and biosecurity measure to ensure prevention of diseases from entering the farm
- ✓ Systematic and strategic deworming of dams and other adult stock and young stock >3 months must be delivered to minimize parasitic disease burden (e.g. against round worms, fasciolosis, ectoparasites).
- ✓ Deworm pregnant dams before 3 weeks and at parturition to avoid intrauterine and via milk transmission some parasites like ascaris.
- ✓ Check rectal temperature of the sick calf and if there is fever; animal health professional must treat the calf with the following medications
    - Give fever medication – flunixin meglumine (1 mg/kg IV every 12-24 hrs)
    - Give diarrhea medication – Kaopectate or Pepto-Bismol
    - If failure of passive transfer of immunity (inadequate colostrums) is suspected, give normal bovine plasma 20-80 ml/kg
    - Rehydrate the animal with ORS (homemade or commercial)
    - If bacterial infection (collibacillosis, salmonellosis, or clostridium) are suspected give parenteral administration of ceftiofur (2.2 mg/kg IM or SC every 12h) and amoxicillin or ampicillin (10 mg/kg IM every 12h)--or sulfonamides (25 mg/kg IV or IM every 24h) for treating calves with diarrhea and systemic illness. If there is no systemic illness, no antibiotic treatment is needed

- If protozoan infection (coccidiosis, Cryptosporidium) is suspected, give either of Decoquinte, Monenzin sodium, Amprolium, Sulfa-antibitics, or SMZ-TMP tablet based on the tentative diagnosis
- If viral (rotavirus, corona, BVD virus) infection is suspecte d, give only
- supportive fluid, provide good food and rest the sick calf

Attention:

- **Don't treat sick calves by yourself if you are not an animal health professional**
- **Don't give anthelmintics to early pregnant dams**
- **Avoid using antibiotics and anthelmintics purchased from uncertified sources (forged drugs)**

Action 5: Laboratory confirmation of diseases causing agents

Note:

- Appropriate samples for specific identification must be taken by veterinary health expert. A veterinarian caring for the sick individuals must always take samples from sick animals for the purpose of confirmatory diagnosis
- Samples must be properly labeled, kept in appropriate preservative or maintained in the proper cold chain until they reach laboratory.
- Samples should be stored locally only temporary until they are transported to the laboratory

Do the followings

- ✓ Collect at least 10 ml of blood from jugular vein (alternatively from tail vein underneath the tail) in the neck region using a vaccutainer tube (fill 2/3 of the tube). Clean the skin at the site of penetration using alcohol/savalon swab

- ✓ Always label each sample (date and identification code). Keep the tube slightly tilted at room temperature overnight (or for at least 2 hrs) to harvest serum if this is the purpose
- ✓ Serum samples should be decanted into a cryovial, labeled and kept in deep freezer at -21 ^{0}C until analysis
- ✓ Fecal samples should be directly taken from the rectum using glove and kept in a universal container. Depending on the protocol, a preservative can be used for the feces
- ✓ Follow instructions of the laboratory technologist for taking, storing and transporting other samples (please refer to SOP for laboratory procedures in identification of diseases causing agents)
- ✓ Nasal swabs must be taken aseptically in a container designed for this.
- ✓ Skin scrapings should be taken using a scalpel blade right at the periphery of the lesion (not from the middle) scrapped until a healthy tissue is visible. Place skin scrapings in appropriate preservative as per the instruction of the laboratory technologist.
- ✓ Samples that require exporting, in case of absence of proper laboratory facility locally, should be temporarily stored at the National Animal Health and Diagnostic Center at Sebeta

Pre-requisites: Vaccines for common and prevalent diseases, anthelmintics, ORS, antibioticsand other preparations, detergent, disinfectants, vaccination and treatment equipment must be available based on public and private animal health service delivery posts/clinics.

INTERVENTION 2: PREVENTION AND CONTROL OF DISEASES CAUSING PNEUMONIA IN CALVES

Description of the intervention:

Pneumonia occurs primarily in housed calves over 2 months of age.

It is caused by infection with respiratory viruses and inadequate ventilation and may accounts for about 15% of calf mortality from birth to 6 months of age.

Clinical disease can usually be avoided if proper attention is paid to overcrowding, inadequate ventilation and air movement and mixing of age groups.

Calves that have suffered from scour are more likely to develop pneumonia later in life. Cold weather stress, poor housing and poor hygiene expose calves to pneumonia. Adequate and quality colostrum also protects calves against pneumonia.

The objective of this intervention is to introduce prevention, control and management actions to reduce calf mortality due to pneumonia.

Skill Capacity building:: awareness creation, training and demonstration related to prevention of diseases of calves causing pneumonia, management of pneumonic calves will be given to extension agents and farmers.

Inputs and delivery mechanisms: The health post need to be strengthened with man power, equipment and supplies. The following list of materials and supplies are required.

- Vaccine: IBR vaccine, Pasteurella vaccine, Manheima vaccine
- Supportive medications: Flunixin meglumine (Banamine); ringers lactate
- Antibiotics: Penicilline, Oxytetracycline, Florfenicol, and Tylosin
- Antiseptics: Savalon, Alcohol, Detergents, and Bleach
- Materials/equipment: protective clothing, vaccination and treatment syringe and needle; sprayer, cotton, gauze, face mask, gloves, laboratory supplies for collecting serum, blood, tissue and other samples.

Materials could be procured through public or private animal health service delivery posts/clinics. Mobile services to villages could be one efficient means of delivery of services.

Action 1: Vaccination

Note:

- All calves must have one 3.5 L of colostrum within four to six hours of birth to receive adequate immunity. Calves that are not given enough antibodies at birth are at increased risk for pneumonia and scours throughout the entire growing period.
- Calf pneumonia is a major problem in dairy and beef herds. It is a multifactorial disease, and the most common post-mortem diagnosis in calves between one to five months of age. Agents of respiratory infection in calves include:
  - *Mannheimia haemolytica*, *Haemophilus somnus*, Infectious Bovine Rhinotracheitis (IBR), bovine Respiratory Syncytial Virus (RSV) and Parainfluenza III Virus (PI3), along with many other bacteria and mycoplasma species and viruses.
- Most calves that have pneumonia will have a fever and a rapid respiratory rate (over 60 per minute)

Do the following:

- Ensure transfer of passive immunity through adequate and timely provision of colostrums (**refer SOP for colostrums feeding**)
- Vaccinate all calves at 8 weeks of age for Pasteurellosis, IBR and if available parainfluenza.
- Vaccinate the breeding female 3-4 weeks prior to parturition to strengthen passive transfer of immunity through colostrums.
- Follow instruction by the manufacturer for vaccination (dose and site of injection) and for adoption refer details in ***Action 1 of Intervention 1 described for Diarrhea***.
- Don’t inject needle in dirty area otherwise it might become infected
- Don’t vaccinate pregnant cows/heifers in the first trimester (first 3 months of pregnancy)
- Don’t vaccinate unhealthy and wet animals

- Don’t wipe injection sites with disinfectant , this can reduce its effectiveness of the vaccine
- Don’t give other medication at the time of vaccination
- Don’t give Banamine or Flunix to a calf IM (give only IV) as it cause pain and severe muscle damage

Action 2: Isolation and quarantine

Note:

- Inadequate ventilation, crowding, commingling of animals from various farms (feedlot or sale barn situations), and other stresses have all been associated with pneumonia.
- Environmental factors include low environmental temperatures and high humidity and poor ventilation and also direct draughts onto calves themselves.

Do the followings:

- Separating the sick calf from the rest of the herd and provide sufficient attention to the sick calf; this prevent the spread of the diseases.
- Deworm pregnant animals before 3 weeks and at parturition
- Systematic and strategic deworming of adult stock and young stock >3 months, (e.g. against lung worms)
- Perform the following during isolation and quarantine of the pneumonic calf:
- Avail a separate, dry and clean barn for isolating sick and newly introduced animals (newly purchased from other farms)
- Properly care and seek professional help for treatment of isolated animals
- Don’t mix sick and apparently healthy animals in the isolation pen
- Don’t delay to seek professional help for cases of pneumonia
- Don’t treat sick young animals yourself if you are not an animal health professional
- Avoid to use antibiotics and anthelmintics purchased from uncertified sources

Action 3: Prevention and Treatment of sick animals

Note:

- A calf must grow one ~1/2 kg per day to maintain an adequate immune system.. Calves must have 5 to 7 Liters of milk per day, fresh water and starter feed to be healthy. Do not overlook the simple fact that calves must grow to be healthy, and in many situations we find inadequate nutrition to be a major cause of calf disease
- One cause of pneumonia in young calves is fluids going into the lungs via the windpipe (trachea). The first feeding of colostrum can cause problems if the feeding rate is faster than swallowing rate. If colostrum is bottle fed it is important to use a nipple that matches the calf's ability to swallow.

Do the followings:

- Make sure colostrums is properly fed to the newborn if it is not sucling
- Viral and bacterial pneumonia spread fast between animals. Hence it is essential to immediately isolate and treat pneumonic calves
- Seek professional assistance if a calf shows signs of pneumonia (acute depression, lethargy and loss of appetite, separation from the remainder of the group, increased respiratory rate with an abdominal breathing and fever)
- Long acting oxytetracycline plus vitamins A, D, and E to all calves in the affected age group is the best antibiotic treatment
- Oxytetracycline is given at a dose rate of 10 mg/kg/day of non-long-acting product, or 20 mg/kg once of the long-acting product. Alternatively give Florfenicol (20 mg/kg, every 48 hr), or Tylosin (10–20 mg/kg, once to twice daily). Always read instruction for prescriptions before giv ing treatment
- Flunixin meglumine (1mg/kg IV every 24hrs) can be given in conjunction with antibiotic therapy for control of endotoxemia and inflammation.
- Therapy should continue for at least 72 hr after body temperature has returned to normal. Duration of treatment usually is 4–5 days.

- Whenever possible, treatment should be based on laboratory confirmation, especially in herd or flock outbreaks, when valuable animals are involved, or in acute or chronic cases when initial therapeutic attempts have failed
- Watch claves at the time of milk feeding for signs of pneumonia. Don't keep young animals without attention for too long if you see the signs of pneumonia
- Don't use antibiotics purchased from uncertified sources (forged drugs)
- Don't treat pneumonic animals yourself unless you are animal health professionals. Most drug resistance occurs due to unprofessional application of antibiotics
- Don't give Banamine or Flunix to a calf IM (give only IV) as it cause pain and severe muscle damage

Action 4: Laboratory Confirmation of diseases causing agents

Note:

- Samples must be collected to confirm causative agents of diseases
- Appropriate samples for specific identification must be taken by veterinary health expert. A veterinarian caring for the sick individuals must always take samples from sick animals for the purpose of confirmatory diagnosis
- Samples must be properly labeled, kept in appropriate preservative or maintained in the proper cold chain until they reach laboratory.
- Samples should be stored locally only temporary until they are transported to the laboratory

Do the followings

- ✓ Collect at least 10 ml (2/3 of the tube) of blood from jugular vein in the neck region or tail using a vaccutainer tube Clean the skin at the site of penetration using alcohol swab
- ✓ Always label each sample (date and identification code). Keep the tube slightly tilted at room temperature overnight (or stand for at least 2 hrs) to harvest serum if this is the purpose

- ✓ Serum samples should be decanted into a cryovial, labeled and kept in deep freezer at -21 ^{0}C.
- ✓ Collect about 5 g of fecal sample directly from the rectum using glove and keep in a universal container. Depending on the protocol, a preservative can be used for the feces
- ✓ Nasal swabs must be taken aseptically in a container designed for this.
- ✓ Follow instructions of the laboratory technologist for taking, storing and transporting other samples (***refer to SOP for laboratory procedures in identification of diseases causing agents***)
- ✓ Samples that require exporting should be temporarily stored at the National Animal Health and Diagnostic Center at Sebeta

Pre-requisites: Vaccines for common and prevalent diseases, anthelmintics, antibiotics and other preparations, detergent, disinfectants, vaccination and treatment equipment must be available based on public and private animal health service delivery posts/clinics.

4.3. REFERENCES/RESOURCES FOR FURTHER READING

Aleme, A., and Z. Lemma. 2015. Contribution of Livestock Sector in Ethiopian Economy. *Advances in Life Science and Technology* 29:2224-7181.

Behnke, R., and F. Metaferia. 2011. The contribution of livestock to the Ethiopian economy-Part II. IGAD Livestock Policy Initiative Working Paper No. 02 – 11.

Catley, A., B. Admassu, G. Bekele, and D. Abebe. 2014. Livestock mortality in pastoralist herds in Ethiopia and implications for drought response.*Disasters* 38(3): 500−516.

Fentie, T., Temesgen, W., Melaku, A., Assefa, G., Tesfaye, S., et al. 2016. Assessment of young stock mortality in major livestock production systems of Ethiopia. Feed The Future, The U.S. Government's Global Hunger and Food Security Initiative, USAID.

Hailu, D., G. Mieso, A. Nigatu, D. Fufa, and D. Gamada. 2006. The effect of environmental factors on pre-weaning survival rate of Borana and Arsi-bale kids. *Small Ruminant Research* 66:291–294.

Inamdar, A. 2012. Calf mortality: Causes and controls. Indian Veterinary Research Institute, Bareilly, India.

MoA (Ministry of Agriculture). 2014. Livestock Master Plan (LMP), Roadmaps for growth and transformation. Livestock develo pment sector.MOA, Addis Ababa.

Moran, J. B. 2011. Factors affecting high mortality rates of dairy replacement calves and heifers in the tropics and strategies for their reduction.*Asian-Australasian Journal of Animal Science* 24 (9): 1318–1328.

NABS (Netherland-African Business Council) 2010. Livestock in Ethiopia and opportunity analysis for Dutch investment. Fact sheet: Livestock Ethiopia.

Negassa, A., S. Rashid, and B. Gebremedhin. 2011. Livestock Production and Marketing. ESSP II, Working Paper 26. International Food Policy Research Institute/ Ethiopia Strategy Support Program II, Addis Ababa, Ethiopia.

Radostitis, O. M. 2005. *Herd health: Food animal production medicine*. 3rd ed. WB. Saunders Company.

NATIONAL YOUNG STOCK MORTALITY REDUCTION PACKAGE

Standard Operation Procedure (SOP) for pastoral/agro-pastoral system

MAY 30, 2018

Addis Ababa, Ethiopia

VII. CHAPTER V: CALF MORTALITY REDUCTION PACKAGE FOR PASTORAL LIVESTOCK PRODUCTION SYSTEM

Introduction

Calf mortality in pastoral / agro pastoral areas is the most important factor affecting performance of livestock production and inflicts major socio- economic losses s. The annual birth to weaning mortality of young stock and the premature death (abortion and stillbirth) was reported in the range of 26-29.2 % and 17.4-21.2% in cattle, respectively. Poor husbandry / management practice and inadequate veterinary services are the major factors favoring the expansion of calf diseases that cause morbidity and mortality. Among the diseases, diarrhea and respiratory disorders (pneumonia) are the most important.

Therefore, in order to minimize the losses of livestock production in pastoral / agro pastoral areas, the causes of animal morbidity and mortality and the associated risk factors like diarrhea, pneumonia and other related agents need to be prevented and appropriate control measures implemented through this package of interventions.

Objective of the package

- ✓ To describe the major predispose factors and causes of young stock mortality in calves.
- ✓ To introduce integrated interventions for the control of the major predisposing factors and diseases that causes morbidity and mortality in calves in the pastoral areas of the country.

5.1 MANAGEMENT / HUSBANDRY RELATED INTERVENTIONS

INTERVENTION 1: PRENATAL CARE OF THE DAM

Description of the intervention: the objective of this intervention is to give the necessary care tothe pregnant dam during the last trimester of pregnancy and at parturition. Feeding during the last trimester (energy, protein, Ca, Mg, Vit A); retaining the mother in a comfortable and safe area during 1 week before pending parturition and 1 week after parturition and providing a separate clean and dry area.

Skill and knowledge capacity building: for EAs, CAHWs and pastoralist/agro pastoralist on preparation of Urea-Molasses Blocks, Multi-nutrient blocks, Urea treated crop residues, and production of improved forage.

Inputs and delivery mechanisms: Urea-Molasses lick as a business initiative (with credit availability, tax relief, provision of land for the business etc.), Potential cooperative initiative for production at minimized cost, Training and awareness creation in areas unlikely to easily adopt the technology, Urea treatment of crop residues, Targeted marketable mineral lick (Limestone, salt block)

Action 1: Feeding the dam during the last trimester (after six months of pregnancy)

Note:

- ☞ Management decisions made during the last trimester of pregnancy are critical and can influence success at calving, survival of the newborn and profitability of the farm.
- ☞ Underfeeding cows prior to calving could increase calf scours and mortality and reduce calf survival

- ☞ Ideally, most cows should enter the calving season with a body condition score (BCS) of 5 (on a 1 to 9 scale). If the cows are thinner than desired, it is advisable to alter the plane of nutrition to add body condition prior to calving.

Do the following:

- Supplementurea molasses blocks/licks during the last trimester of pregnancy with ample amount of water to enable the dam to get the vital minerals and vitamin that can help to minimize the occurrence of dystocia(**Use ESGPIP technical bulletin No.1 as a reference)**
- Supplement Acacia pods or similar high protein feed resources available in the area
- Give priority to these animals in the supply of whatever better quality feed available
- Make sure young ruminants less than 6 months of age and other species of non-ruminant domestic animals e.g. donkeys don't get access to Urea-Molasses licks
- Don't keep the lick in wet and moist place
- Don't feed ground or water dissolved lick
- Give priority in the access to area closures/reserves for grazing and supply of harvested feed e.g. hay;

Action 2: Retaining the dams around the homestead

Note: retaining the dams near the homestead is important to attend to calving difficulties and assist weak calves

Do the following:

- Don't send pregnant animals close to delivery (one week before expected date of delivery) long distance with the rest of the herd for the purpose of grazing. Retain the pregnant animal in a safe and comfortable place at least one week before expected parturition and another one week after parturition.
- Provide a separate clean and dry area /dry enclosure

- Provide plenty of fresh clean water immediately after delivery, supplement with moderate amount of good quality roughage for the first two days and gradually introduce 250-300 gm of concentrate per head per day for the remaining days as available
- Continue the lactation supplementation for the dam during the pre-weaning period

Pre-requisites: Accessibility of molasses, Regular monitoring system for implementation sustainability (EAs and CAHWs will be primarily responsible for monitoring the implementation s), Enforcement mechanism.

INTERVENTION 2: NEONATAL CARE OF THE CAL VES

Description of the intervention: the objective of this intervention is to give the necessary care for newborn calves from birth up to 1 months of age. Induction of breathing through clearing the air way and chest massage, navel treatment, improving peripheral circulation, feeding colostrum, providing due attention in case of twin birth.

Skill and knowledge capacity building: for EAs, CAHWs and pastoralists/agro pastoralists on management and care of newborn animals.

Inputs and delivery mechanisms: Plastic bulb pipetter**,** Warm cloth, Milk feeder, Colostrum replacer, foster colostrum in case of dead dam or poor mothering, detergent, antiseptics, clean thread and blade, scale, protective wear for professionals (as deemed necessary),

Action 1: Induction of breathing

Note:

- Survival of the newborn is dependent on the rapid onset of normal, spontaneous respiration.

- In a normal calf if parturition occurs normally, respiration usually commences after 30 seconds of birth, being irregular at first before settling down to 45–60 breaths per minute.
- Once birth is complete, it is important first to ensure that the upper respiratory tract is cleared of fluid, mucus and attached fetal membrane. This can be done with the aid of fingers or, preferably, with a simple suction device (plast ic bulb pipette)

Do the following:

- ✓ Start assisting the dam and the calf after washing your hand or use protective gloves at all times
- ✓ Clean the nostrils from any mucus or fluid by gently squeezing the muzzle
- ✓ Hold the newborn by the hind legs and lift upside down for a few seconds to expel fluid from the lungs (as shown in Fig. 4)
- ✓ Insert a finger into the mouth, or gently pull out the tongue until the newborn produces sound
- ✓ Brisk rubbing or gentle massage of the chest with towels frequently provides the necessary tactile stimulus to stimulate respiration.
- ✓ Provide extra care for premature, weak and small calves or seek help of an animal health professional
- ☒ Don't insert sharp and dirty material into the nostril to induce sneezing as this may cause injury

Action 2: Navel treatment

Note: The umbilicus usually ruptures passively in cattle. It is hence important to handle it properly.

Do the following:

- Thoroughly wash your hands with soap and water
- Tie (litigate) the umbilical cord at 5 cm (approximately th ree fingers) length with clean string or thread (Fig. 5a)
- Cut the remaining part of the umbilical cord and dip or rub the stump with antiseptics (with 1% Iodine tincture) (Fig. 5b) or apply antibiotic spray (Fig. 5c)

- Carefully observe the umbilicus to dry and heal over the next few days. If it has not dried and sealed off, immediately seek the help of an animal health professional

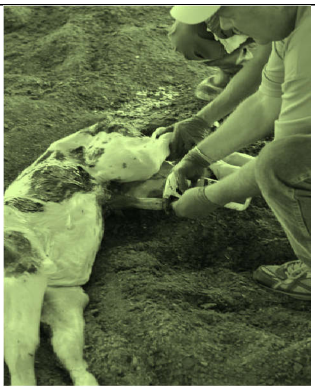	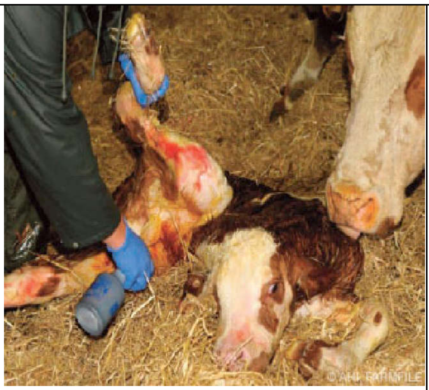	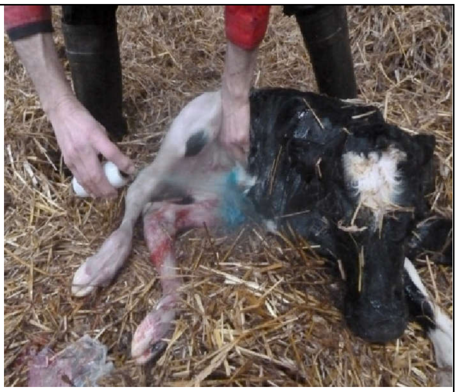
Fig. 5a. Tie the umblical cord	Fig. 5b. Dip the navel stump in iodine tincture	Fig. 5c. Spray the navel stump with antibiotic spray

Action 3: Improving thermoregulation

Note:

The need for improving thermoregulation under the pastoral/agro-pastoral setting may not be a serious problem under normal warm temperatures in these areas. Action in this regard may sometimes be necessary.

Do the following:

- After the dam gives normal birth, dry the entire body of the newborn with a clean cloth or allow the dam to lick the calf.
- Move the newborn and its dam to a dry and clean place with good bedding
- If the newborn is too weak to stand, provide adequate colostrum and allowed it to stay with the dam.
- Never pour cold water on the body of the calf after birth.

Action 4: Feeding colostrum

Note:

☞ Colostrum is critical to survival of the newborn calf.

- ☞ The immune system of newborn calves is not completely developed and the immunoglobulins in colostrum contribute a substantial component of the immune protection in newborn calves.

Do the following:

- o Make sure the newborn suckles the dam immediately after birth or within 6 hrs. Make sure free suckling for at least 4 days

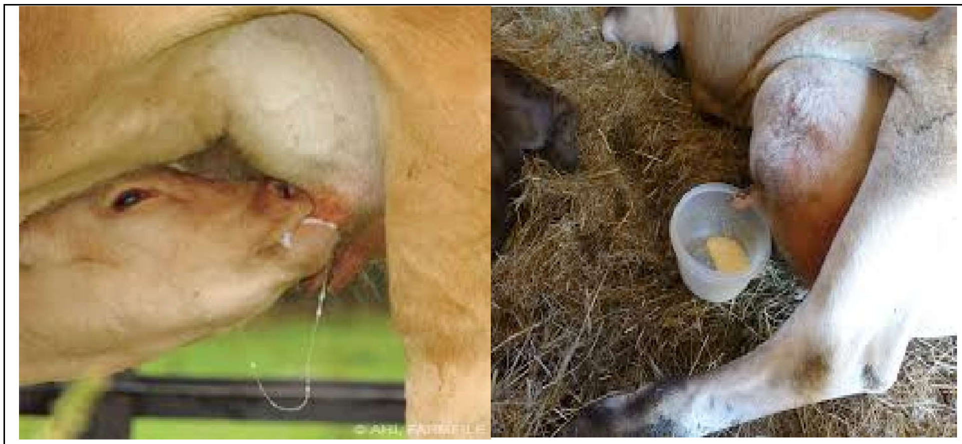

Assist the calf to suckle or feed colostrum by milking within six hours of birth

- o Check if the dam is producing sufficient colostrum by test-milking each of the four teats
- o If the newborn is not suckling for any reason, milk the dam and feed using milk feeding devices such as nipple feeders, stomach tube, or catheter syringe.

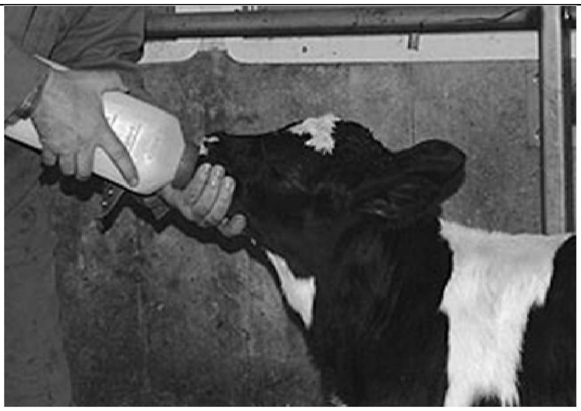

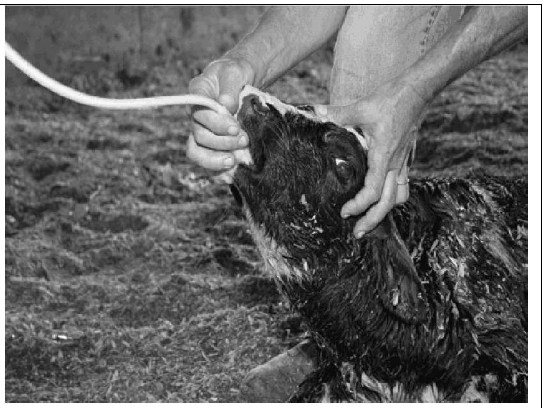

Feeding the newborn calf using milk feeding device or stomach tube

- If the dam does not produce enough colostrum, feed colostrum from a contemporary foster mother or colostrum substitute as last resort (*preparation: 1 egg beaten+400ml fresh warm water+600ml of whole milk+1tea spoonful of oil*)
- Do not delay feeding colostrum feeding beyond 6 hrs after birth
- Avoid competition by humans for the colostrum where the practice is common

Pre-requisites: Accessible disinfection service, Regular monitoring system for implementation sustainability (EAs, CAHWs will be primarily responsible for monitoring the implementation s), Enforcement mechanism

INTERVENTION 3: PRE-WEANING FEEDING MANAGEMENT OF CALVES

Description of the intervention: the objective of this intervention is to give the necessary nutritional supply to the calf during the pre-weaning period from week one to 12 months of age. Appropriate milk feeding, preferential and separate feeding;

Skill and knowledge capacity building: for EAs, CAHWs and train pastoralists/agro pastoralists on the cause of calf mortality due to poor feeding

Inputs and delivery mechanisms: Training and awareness creation in pastoral areas in order to easily adopt the technology,

Action 1: Appropriate milk feeding

Note:

Milk contains all the necessary elements to meet the demand by the newborn calf. It is thus essential that the newborn consumes adequate milk during this period.

Do the following:

- Make sure the calf suckles properly and freely
- Check whether the dam is producing sufficient milk by test milking all the four teats
- If the dam does not produce enough milk or in case of twin births resulting in shortage of milk, implement foster milk feeding
- If the newborn is not suckling for any reason, milk the dam and feed using milk feeding devices such as nipple feeders, catheter syringe, or stomach tube
- Place good quality hay, water and protein supplement (such as high quality lush forage) near the calf to encourage early eating and drinking around two weeks of age.

Action 2: Preferential and separate feeding

Note:

- ☞ As the calf grows, its nutrient requirement also grows. However, it can be difficult to reach a maintenance feeding level when there are competitors.
- ☞ The calf requires a separate extra feeding care that can be provided as follows

Do the following:

- Always use clean milk feeders and other feeding and watering equipment
- Use only freshly prepared feed daily
- provide supplementary feeding during drought season
- Don’t give concentrate before feeding roughage
- Don’t over feed concentrate to calves
- Don’t feed moldy feed to the young
- Don’t give urea treated roughage before four months of age

Pre-requisites: Accessibility of supplementary feeds, Regular monitoring system for implementation sustainability (EAs and CAHWs will be primarily responsible for monitoring the implementation)

INTERVENTION 4: REPRODUCTIVE MANAGEMENT

Description of the intervention: Ensuring the heifer or cow is big enough at calving is important. Heifers should have reached at least 60-65% of their mature body weight at breeding. The purpose of this intervention is to arrange the mating and calving season to match with forage availability and low disease burden to maximize calf survival. Controlled mating and mating by selecting appropriate sires or by estrus synchronization technique would help calving during the planned season and avoid oversized fetuses that lead to dystocia.

Skill and knowledge capacity building:

- Skill training and awareness creation on controlled breeding and estrus synchronization to extension agents, CAHWs and pastoralists/agro-pastoralists is required.
- Training on heat detection will also be given to pastoralists.
- Demonstration on application of castration of males unwanted for breeding and separation of breeding males from the herd at times when breeding is not wanted

Inputs and delivery mechanisms: Input for estrus synchronization (eg hormones, pregnancy diagnosis kit), burdizzo are to be provided by the public livestock development office.

Action 1: Control of mating/ calving

Note:

- Calf survival depends on the dams ability to provide care including adequate supply of milk and this depends on the season which determines feed availability and cold stress
- The weather condition also directly affects the calf performance aggravating the occurrence of pneumonia,
- Thus it is important to match calving with favorable seasons

Do the following

- Identify the most appropriate season for maximum calf survival
- Separate herding of the breeding males outside of the identified breeding season
- Introduce estrus synchronization(agro pastoralist) protocol for cattle
- Appropriate selective breeding based on local practice
- Prevent unwanted mating using castration
- Select calves with larger body size for breeding. Make sure that the pelvic size of pre breeding heifers is greater than 150 square cm
- Ensure heifers are mated at the proper age (12-18 months)
- Don't allow mating between related animals
- Do not sell all large calves with large body size to avoid genetic erosion/negative selection (retain the good ones for breeding)
- Identify the most appropriate season for maximum calf survival in consultation with farmers and based on research results if studies relevant for the locality or similar agroecology are available
- Identify the breeding male or use artificial insemination (AI)
- Castrate bulls unwanted for breeding starting from 2 years of age
- Separate breeding male/ young bulls outside of the identified breeding season
- Introduce and strictly follow estrus synchronization protocol of the MoAL for cows/heifers
- use teaser bulls for heat detection
- Select calves with larger body size for breeding. Make sure that the pelvic size of pre breeding heifers is greater than 150 square cm

- Ensure heifers are mated at the proper age (12-18 months)

- Avoid breeding malpractices such as opening the cervix with stick/ any foreign materials or follow septic procedure.

Pre-requisites:

- Strictly following estrus synchronization protocols is required for successful implementation of the intervention
- Support from Research centers and universities required to provide training on estrus synchronization and conduct research to refine estrus synchronization protocols
- Regular monitoring and evaluation system is required as described for the other interventions.

5.2 HEALTH RELATED INTERVENTIONS

INTERVENTION 1: PREVENTION AND CONTROL OF DISEASES CAUSING DIARRHEA IN CALVES

Description of the intervention: Acute diarrhea accounts for approximately 75% of the mortality of dairy calves under 3 weeks of age.

The main objective of this intervention is to introduce prevention, control and management strategies to reduce calf mortality due to diarrhea. This requires several steps among which vaccination of calves, vaccination of the cow to boost passive transfer of immunity, Adequate and timely Colostrum feeding, availing isolation and quarantine area, rehydration of scouring calves and seeking help of animal health professionals for treatment of sick calves

Diarrhea is not always the result of infectious agents. It can also be the result of stress, poor management and poor nutrition.

Neonatal diarrhea could be caused by nutritional disorders, viruses or bacteria; digestive upsets leading to scours are generally a major cause of death in young calves.

The most important pathogens associated with calf diarrhea are enterotoxigenic *Escherichia coli*, rotavirus, corona virus, *Cryptosporidia* sp. *Salmonella* sp. and Coccidiosis (*Eimeria* sp.).

- *E. coli* may affect calves as young as 16 to 24 hours of age but it is most common in calves 1-7 days old.
- *Clostridium perfringens* type C is most common in calves 1-3 weeks of age. This bacterium is a soil born organism and causes calf scours on many unhygienic farms.
- Salmonella typhimurium is the most common cause. Calves are usually affected at 1-7 days of age. The source of Salmonella infection in a herd can be from other cattle, birds, cats, rodents, water supply or human carriers.
- Rota virus infection can occur in calves as young as 0-28 days old; however, most outbreaks occur when calves are 1-6 days old
- Coronavirus can occur in calves 0-28 days old but it is most common in calves of 7-10 days of age. The virus is not as resistant to degradation as Rotavirus. Adult cows shed the virus in feces and contaminate calving areas. Rota- and coronavirus are hardy and survive for months in moist and cool conditions
- Bovine viral diarrhea (BVD) virus infection is common in young calves. Diarrhea begins about 26 hrs to 3 days after exposure and may persist for quite a long time
- Cryptosporidium is most common in calves 1-3 weeks of age. As a rule, cryptosporidium is detected in combination with coronavirus, rotavirus, and/or E. coli.
- Coccidiosis occur in calve 1 week of age. However; outbreaks associated with stress, poor sanitation, over-crowding, or sudden changes in feed in calves 3-4 weeks of age and older have been reported.

Skill Capacity building: awareness creation, training and demonstration related to prevention of diseases of calves causing diarrhea, management of diarrheic calves, preparation of rehydrating solutions locally will be given to extension agents and farmers.

Inputs and delivery mechanisms: The following inputs are required

- Vaccine: bovine E coli vaccine (K99 bacterin), bovine rotavirus vaccine and bovine corona virus vaccine
- Antiparasites: Albendazol, Tetramizol,, Fenbendazol, Decoquinate, Sulfadimidin, Amprolium, Monenzine sodium
- Antibiotics: Penicillin (also as Penstrep), Neomycine, Spectinomycine, , SMZ-TMP (Bacterium) tablets
- Supportive treatments: Oral rehydration salt (ORS), flunixin meglumine (Benamine), Normal bovine plasma, Dextrose saline, normal saline, ringers lactate
- Antiseptics: Formalin (10%), Virocyl, Bleach, Savalon, Alcohol (denatured), Detergents

Materials/equipment: protective clothing, vaccination and treatment syringe and needle, sprayer, cotton, gauze, gloves

Action 1: Vaccination

Note:

- Vaccination helps the calf to cope with diseases challenges in the new environment.
- Prevention of diarrhea can be made indirectly by vaccinating the pregnant dam or by directly vaccinating the young.
- Vaccinate the dam close to the time of parturition and then timely provide adequate colostrum to the calf to give protection for few months until the calf itself is able to produce antibodies.

- The age at which vaccination is made for the calf may vary for different diseases. Usually colostrum must be fed within few hours of birth while vaccination of calves is made after eight weeks of age.

Do the followings:

- Vaccinate the breeding female before pregnancy to strengthen passive transfer of immunity through the colostrums right after delivery
- Follow the instructions by the manufacturer for vaccinations
- Don't vaccinate pregnant cows/heifers at early stage of pregnancy and calves before six months of age
- Ensure passive immunity through adequate and timely provision of colostrums (***refer to the SOP for colostrum feeding in this manual***)
- Vaccinate all calves above 8 weeks of age for bovine *E coli* vaccine (K99 bacterin), bovine rotavirus vaccine, corona virus, and BVD vaccine
- Vaccinate all calves at six months of age for LSD and FMD
- Vaccinate the pregnant female around one month before parturition with bovine E coli vaccine (K99 bacterin), bovine rotavirus, corona virus vaccine, and BVD vaccine to strengthen passive transfer of immunity through colostrum
- Preferably use 2ml, IM injection (side of the neck) of ***Rotavec® Corona*** vaccine that can protect against Rota, Corona and E coli
- Follow the instruction by the manufacturer for vaccination and withdrawal

- ***Generally, the following steps might apply for vaccination***
    - Read the labels on the vaccine vial every time you are about to vaccinate even if you know the label by heart
    - Record in a notebook which calf you are vaccinating, the date and the vaccine batch to avoid mistakes of revaccination
    - Use the proper syringe and a needle (18 gauge and at least 2 cm long)
    - Disinfect the needle between vaccination if you are not using disposable needles

- Inject the needle on either side of the neck, pinch the skin and inject 2 ml vaccine parallel to the skin (SQ) and repeat as prescribed for the vaccine
- Make sure you store the vaccines properly usually refrigerated at +4 degrees
- Unused vaccine must be discarded. Partially used vaccines will be contaminated and are dangerous for the animal if used
- If you inject yourself mistakenly, seek help of a physician immediately
- Don't inject needle in dirty area otherwise it might become infected
- It is good to replace the vaccination needle once for every 20 animals
- Don't vaccinate pregnant cows/heifers in the first trimester (first 3 months of pregnancy)
- Don't vaccinate unhealthy and wet animals
- Don't wipe injection sites with disinfectant, this can reduce the effectiveness of the vaccine
- Don't give other medication at the time of vaccination

Action 2: Isolation and quarantine

Do the following

- Observe calves during they are suckling to identify scouring animals as early as possible for remedial treatment
- Avail a separate dry and claan enclosure for isolating sick and newly introduced animals
- Properly care and seek help of animal health professionals/CAHWs for treatment of isolated animals
- If a calf is dead, properly dispose by burying or burning following the recommendation of animal health expert (awareness creation on disposal/training (one disposal pit/village may be required)
- Don't mix sick and apparently healthy animals.
- Don't delay to seek professional assistance for cases of diarrhea

Action 3: Rehydration of diarrheic calves

Note:

- It is essential to replace lost fluid due to diarrhea as a first measure to save the life of the calf.
- The critical to remember that dehydrated calves need 6.5 to 8 liters of fluid daily to correct the dehydration. They also need an adequate caloric intake for maintenance and growth

Do the following

- ✓ Provide oral rehydration salts (readymade/home prepared) imme diately upon detection of diarrhea (10% of BW three times/day) until diarrhea stops
- ✓ Prepare homemade rehydration solution as follows

Solution 1

- 6 tablespoonfuls of sugar
- ¼ tablespoonfuls of salt
- Mix into 1 liter of clean water and use 7 or 8 liters every 24 hours

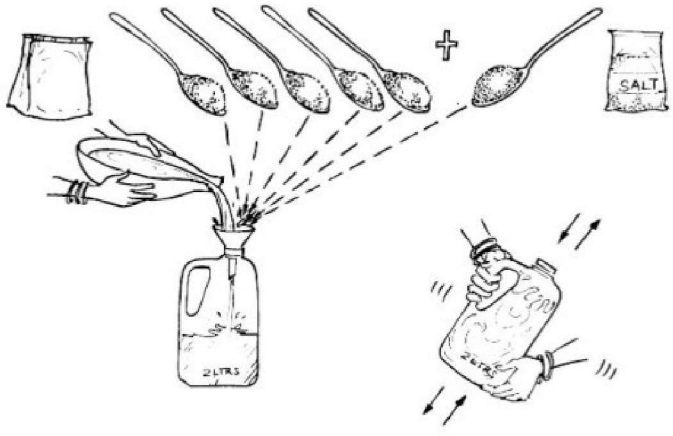

Solution 2

- ¼ tablespoonfuls of salt
- ¼ tablespoonfuls of baking soda (optional)
- 6 tablespoonfuls of sugar
- 2 tablespoonfuls of lemon juice (optional)
- Mix into 1 liter of clean water and use 7 or 8 liters every 24 hours

- ✓ . Don’t trek sick and diarrheic calves (Rest diarrheic calf until recovery)
- ✓ Don’t stop provision of ORS before diarrhea stops
- ✓ Oral rehydration salt (ORS) is a supportive treatment hence don’t consider ORS as a final and curative treatment
- ✓ Don’t give antibiotics to treat scours resulting from over-feeding or digestive upsets. Blood scours (mostly caused by coccidian or other microorganisms) require veterinary treatment and management changes to improve hygiene.

Action 4: Prevention and Treatment of sick animals

Note:

- Before treating a scoring calf, it is essential to determine why it is scouring. Take the calf's temperature using a rectal thermometer. If body temperature is above the normal range (38.5-39°C), fever medications such as Flunixin meglumine and antibiotics must be given.

Do the followings:

- ✓ Systematic and strategic deworming of dams and other adult stock and young stock >3 months must be delivered to minimize parasitic disease burden (e.g. against round worms, ectoparasites).
- ✓ Deworm pregnant dams before 3 weeks and at parturition to avoid intrauterine and via milk transmission some parasites like ascaris.
- ✓ Check rectal temperature of the sick calf and if there is fever; animal health professional must treat the calf with the following medications
    - Give fever medication – flunixin meglumine (1 mg/kg IV every 12-24 hrs)
    - Give diarrhea medication – Kaopectate or Pepto-Bismol
    - If failure of passive transfer of immunity (inadequate colostrums) is suspected, give normal bovine plasma 20-80 ml/kg
    - Rehydrate the animal with ORS (homemade or commercial)
    - If bacterial infection (collibacillosis, salmonellosis, or clostridium) are suspected give parenteral administration of ceftiofur (2.2 mg/kg IM or SC every 12h) and amoxicillin or ampicillin (10 mg/kg IM every 12h)--or

sulfonamides (25 mg/kg IV or IM every 24h) for treating calves with diarrhea and systemic illness. If there is no systemic illness, no antibiotic treatment is needed

  - If protozoan infection (coccidiosis, Cryptosporidium) is suspected, give either of Decoquinte, Monenzin sodium, Amprolium, Sulfa-antibitics, or SMZ-TMP tablet based on the tentative diagnosis
  - If viral (rotavirus, corona, BVD virus) infection is suspected, give only supportive fluid, provide good food and rest the sick calf

- Don’t treat sick young animals yourself if you are not an animal health professional /CAHW
- Don’t give anthelmintics to early pregnant animals
- Don’t use antibiotics and anthelmintics purchased from uncertified sources (forged drugs)

Action 5: Laboratory confirmation of diseases causing agents

Note:

- Appropriate samples for specific identification must be taken by veterinary health expert. A veterinarian caring for the sick individuals must always take samples from sick animals for the purpose of confirmatory diagnosis
- Samples must be properly labeled, kept in appropriate preservative or maintained in the proper cold chain until they reach laboratory.
- Samples should be stored locally only temporary until they are transported to the laboratory

Do the followings

  - ✓ Collect at least 10 ml of blood from jugular vein (alternatively from tail vein underneath the tail) in the neck region using a vaccutainer tube (fill 2/3 of the tube). Clean the skin at the site of penetration using alcohol/savalon swab

- ✓ Always label each sample (date and identification code). Keep the tube slightly tilted at room temperature overnight (or for at least 2 hrs) to harvest serum if this is the purpose
- ✓ Serum samples should be decanted into a cryovial, labeled and kept in deep freezer at -21 ^{0}C until analysis
- ✓ Fecal samples should be directly taken from the rectum using glove and kept in a universal container. Depending on the protocol, a preservative can be used for the feces
- ✓ Follow instructions of the laboratory technologist for taking, storing and transporting other samples (please refer to SOP for laboratory procedures in identification of diseases causing agents)
- ✓ Nasal swabs must be taken aseptically in a container designed for this.
- ✓ Skin scrapings should be taken using a scalpel blade right at the periphery of the lesion (not from the middle) scrapped until a healthy tissue is visible. Place skin scrapings in appropriate preservative as per the instruction of the laboratory technologist.
- ✓ Samples that require exporting, in case of absence of proper laboratory facility locally, should be temporarily stored at the National Animal Health and Diagnostic Center at Sebeta

Pre-requisites: Vaccines for common and prevalent diseases, anthelmintics, ORS, antibioticsand other preparations, detergent, disinfectants, vaccination and treatment equipment must be available based on public and private animal health service delivery posts/clinics.

INTERVENTION 2: PREVENTION AND CONTROL OF DISEASES CAUSING PNEUMONIA IN CALVES

Description of the intervention:

Pneumonia occurs primarily in housed calves over 2 months of age.

It is caused by infection with respiratory viruses and inadequate ventilation and may accounts for about 15% of calf mortality from birth to 6 months of age.

Clinical disease can usually be avoided if proper attention is paid to overcrowding, inadequate ventilation and air movement and mixing of age groups.
Calves that have suffered from scour are more likely to develop pneumonia later in life.

Cold weather stress, poor housing and poor hygiene expose calves to pneumonia. Adequate and quality colostrum also protects calves against pneumonia.
The objective of this intervention is to introduce prevention, control and management actions to reduce calf mortality due to pneumonia.

Skill Capacity building:: awareness creation, training and demonstration related to prevention of diseases of calves causing pneumonia, management of pneumonic calves will be given to extension agents and farmers.

Inputs and delivery mechanisms: The health post need to be strengthened with man power, equipment and supplies. The following list of materials and supplies are required.

- Vaccine: IBR vaccine, Pasteurella vaccine, Manheima vaccine
- Supportive medications: Flunixin meglumine (Banamine); ringers lactate
- Antibiotics: Penicilline, Oxytetracycline, Florfenicol, and Tylosin
- Antiseptics: Savalon, Alcohol, and Detergents
- Materials/equipment: protective clothing, vaccination and treatment syringe and needle; sprayer, cotton, gauze, face mask, gloves, laboratory supplies for collecting serum, blood, tissue and other samples.

Materials could be procured through public or private animal health service delivery posts/clinics. Mobile services to villages could be one efficient means of delivery of services.

Action 1: Vaccination

Note:

- All calves must have one 3.5 L of colostrum within four to six hours of birth to receive adequate immunity. Calves that are not given enough antibodies at birth are at increased risk for pneumonia and scours throughout the entire growing period.
- Calf pneumonia is a major problem in dairy and beef herds. It is a multifactorial disease, and the most common post-mortem diagnosis in calves between one to five months of age. Agents of respiratory infection in calves include:
  - *Mannheimia haemolytica*, *Haemophilus somnus*, Infectious Bovine Rhinotracheitis (IBR), bovine Respiratory Syncytial Virus (RSV) and Parainfluenza III Virus (PI3), along with many other bacteria and mycoplasma species and viruses.
- Most calves that have pneumonia will have a fever and a rapid respi ratory rate (over 60 per minute)

Do the following:

- Ensure transfer of passive immunity through adequate and timely provision of colostrums (**refer SOP for colostrums feeding**)
- Vaccinate all calves at 8 weeks of age for Pasteurellosis, IBR and if available parainfluenza.
- Vaccinate the breeding female 3-4 weeks prior to parturition to strengthen passive transfer of immunity through colostrums.
- Follow instruction by the manufacturer for vaccination (dose and site of injection) and for adoption refer details in ***Action 1 of Intervention 1 described for Diarrhea***.
- Don't inject needle in dirty area otherwise it might become infected
- Don't vaccinate pregnant cows/heifers in the first trimester (first 3 months of pregnancy)
- Don't vaccinate unhealthy and wet animals

- Don’t wipe injection sites with disinfectant , this can reduce its effectiveness of the vaccine
- Don’t give other medication at the time of vaccination
- Don’t give Banamine or Flunix to a calf IM (give only IV) as it cause pain and severe muscle damage

Action 2: Isolation and quarantine

Note:

- Inadequate ventilation, crowding, commingling of animals from various farms (feedlot or sale barn situations), and other stresses have all been associated with pneumonia.
- Environmental factors include low environmental temperatures and high humidity and poor ventilation and also direct draughts onto calves themselves.

Do the followings:

- Separating the sick calf from the rest of the herd and provide sufficient attention to the sick calf; this prevent the spread of the diseases.
- Deworm pregnant animals before 3 weeks and at parturition
- Systematic and strategic deworming of adult stock and young stock >3 months, (e.g. against lung worms)
- Perform the following during isolation and quarantine of the pneumonic calf:
- Avail a separate, dry and clean barn for isolating sick and newly introduced animals (newly purchased from other farms)
- Properly care and seek professional help for treatment of isolated animals
- Don’t mix sick and apparently healthy animals in the isolation pen
- Don’t delay to seek professional help for cases of pneumonia
- Don’t treat sick young animals yourself if you are not an animal health professional
- Avoid to use antibiotics and anthelmintics purchased from uncertified sources

Action 3: Prevention and Treatment of sick animals

Note:

- A calf must grow one ~1/2 kg per day to maintain an adequate immune system.. Calves must have 5 to 7 Liters of milk per day, fresh water and starter feed to be healthy. Do not overlook the simple fact that calves must grow to be healthy, and in many situations we find inadequate nutrition to be a major cause of calf disease
- One cause of pneumonia in young calves is fluids going into the lungs via the windpipe (trachea). The first feeding of colostrum can cause problems if the feeding rate is faster than swallowing rate. If colostrum is bottle fed it is important to use a nipple that matches the calf's ability to swallow.

Do the followings:

- Make sure colostrums is properly fed to the newborn if it is not suckling
- Viral and bacterial pneumonia spread fast between animals. Hence it is essential to immediately isolate and treat pneumonic calves
- Seek professional assistance if a calf shows signs of pneumonia (acute depression, lethargy and loss of appetite, separation from the remainder of the group, increased respiratory rate with an abdominal breathing and fever)
- Long acting oxytetracycline plus vitamins A, D, and E to all calves in the affected age group is the best antibiotic treatment
- Oxytetracycline is given at a dose rate of 10 mg/kg/day of non-long-acting product, or 20 mg/kg once of the long-acting product. Alternatively give Florfenicol (20 mg/kg, every 48 hr), or Tylosin (10–20 mg/kg, once to twice daily). Always read instruction for prescriptions before giving treatment
- Flunixin meglumine (1mg/kg IV every 24hrs) can be given in conjunction with antibiotic therapy for control of endotoxemia and inflammation.
- Therapy should continue for at least 72 hr after body temperature has returned to normal. Duration of treatment usually is 4–5 days.

- Whenever possible, treatment should be based on laboratory confirmation, especially in herd or flock outbreaks, when valuable animals are involved, or in acute or chronic cases when initial therapeutic attempts have failed
- Watch claves at the time of milk feeding for signs of pneumonia. Don't keep young animals without attention for too long if you see the signs of pneumonia
- Don't use antibiotics purchased from uncertified sources (forged drugs)
- Don't treat pneumonic animals yourself unless you are animal health professionals. Most drug resistance occurs due to unprofessional application of antibiotics
- Don't give Banamine or Flunix to a calf IM (give only IV) as it cause pain and severe muscle damage

Action 4: Laboratory Confirmation of diseases causing agents

Note:

- Samples must be collected to confirm causative agents of diseases
- Appropriate samples for specific identification must be taken by veterinary health expert. A veterinarian caring for the sick individuals must always take samples from sick animals for the purpose of confirmatory diagnosis
- Samples must be properly labeled, kept in appropriate preservative or maintained in the proper cold chain until they reach laboratory.
- Samples should be stored locally only temporary until they are transported to the laboratory

Do the followings

- ✓ Collect at least 10 ml (2/3 of the tube) of blood from jugular vein in the neck region or tail using a vaccutainer tube Clean the skin at the site of penetration using alcohol swab
- ✓ Always label each sample (date and identification code). Keep the tube slightly tilted at room temperature overnight (or stand for at least 2 hrs) to harvest serum if this is the purpose

- ✓ Serum samples should be decanted into a cryovial, labeled and kept in deep freezer at -21 ^{0}C.
- ✓ Collect about 5 g of fecal sample directly from the rectum using glove and keep in a universal container. Depending on the protocol, a preservative can be used for the feces
- ✓ Nasal swabs must be taken aseptically in a container designed for this.

Pre-requisites: Vaccine, anthelmintics, ORS, Antibiotics and other preparations, detergent, disinfectants, protective clothing, vaccination and treatment equipment. Based on public and private animal health service delivery posts/clinics

5.3. REFERENCE/RESOURCES FOR FURTHER READING

AlemuYami. 2007. How to make Urea Molasses Blocks (UMB) and feed to Sheep and Goats. Technical Bulletin No.1. Ethiopia Sheep and Goat productivity Improvement Program. R.C. Merkel (ed.). 10Pp

Behnke, R., and F. Metaferia. 2011. The contribution of livestock to the Ethiopian economy-Part II. IGAD Livestock Policy Initiative Working P aper No. 02 – 11.

Catley, A., B. Admassu, G. Bekele, and D. Abebe. 2014. Livestock mortality in pastoralist herds in Ethiopia and implications for drought response.*Disasters* 38(3): 500–516.

Fentie, T., Temesgen, W., Melaku, A., Assefa, G., Tesfaye, S., et al. 2016. Assessment of young stock mortality in major livestock production systems of Ethiopia. Feed The Future, The U.S. Government's Global Hunger and Food Security Initiative, USAID.

Hailu, D., G. Mieso, A. Nigatu, D. Fufa, and D. Gamada. 2006. The effect of environmental factors on pre-weaning survival rate of Borana and Arsi-bale kids. *Small Ruminant Research* 66:291–294.

Inamdar, A. 2012. Calf mortality: Causes and controls. Indian Veterinary Research Institute, Bareilly, India.

MoA (Ministry of Agriculture). 2014. Livestock Master Plan (LMP), Roadmaps for growth and transformation. Livestock development sector.MOA, Addis Ababa.

Moran, J. B. 2011. Factors affecting high mortality rates of dairy replacement calves and heifers in the tropics and strategies for their reduction.*Asian-Australasian Journal of Animal Science* 24 (9): 1318–1328.

NABS (Netherland-African Business Council) 2010. Livestock in Ethiopia and opportunity analysis for Dutch investment. Fact sheet: Livestock Ethiopia.

Negassa, A., S. Rashid, and B. Gebremedhin. 2011. Livestock Production and Marketing. ESSP II, Working Paper 26. International Food Policy Research Institute/ Ethiopia Strategy Support Program II, Addis Ababa, Ethiopia.

NATIONAL YOUNG STOCK MORTALITY REDUCTION PACKAGE

Standard Operation Procedure (SOP) for Urban/periurban system

MAY 30, 2018

Addis Ababa, Ethiopia

V. CHAPTER III: CALF MORTALITY REDUCTION PACKAGE FOR URBAN AND PERI-URBAN LIVESTOCK PRODUCTION SYSTEM

Introduction

Maintenance and development of a productive cow-herd requires a constant input of new calves. Calf morbidity and mortality are perennial problems that limit this in all countries where cattle are raised. Average mortality in well managed dairy herds in developed countries, is usually between 2 and 4%. In developing countries, under poor management and major disease problems, especially in sub-Saharan Africa, the usual average mortality is 7 to 25%. Most of the illness and deaths occur during the first few weeks of life because of the effects of infection and mismanagement of both the calf and its environment. The main factors include dystocia, parity, birth weight, gestation length, season and year of birth and ratio of calf weight to cow weight. Neonatal calf diarrhea accounting for overwhelming majority of the mortality of dairy calves under 3 weeks of age is one of the most devastating diseases of the dairy industry, and with enzootic calf pneumonia, they are the number one killers of the calf.

According to a more recent investigation under the Feed the Future Project in Ethiopia, dairy calves mortality rates during the pre-weaning period range from 15.3 to 25%, but it could go as high as 32.2%. Mortality rate reaching 67% (including abortion rates and stillbirths), have also been reported in a different study. Age of the calf, colostrum feeding, farm cleanliness, nutritional and management problems were reported to be the major problems associated with high mortality rates. Calf diarrhea and pneumonia have been identified as the leading health problems in newborn calves followed by gastro-intestinal parasites in older calves. Young stock mortality can negatively impact the progress in production and productivity if not handled seriously and urgently.

Objective of the package

The main objective of this package is to introduce and implement calf mortality reduction interventions in the urban and peri-urban dairy production system.

3.1 MANAGEMENT AND HUSBANDRY RELATED INTERVENTIONS

INTERVENTION 1: IMPROVING FARM CLEANLINESS

Description of the intervention: the objective of this intervention is to develop a package for farm cleanliness that contributes to the reduction of calf mortality in the urban and peri-urban system.

Most of diseases causing agents grow and multiply in unhygienic conditions. They get the opportunity to infect animals when the farm's cleanliness is poor.

The intervention package includes aspects of management such as clean and proper flooring, provision of adequate space and comfortable bedding through construction of a proper farming premise. To maintain a clean farm safe for the animals, the floor system must allow for proper drainage and regular disinfection.

Skill and Capacity building:.

- Capacity building of experts and farmers on concepts of farm biosecurity, safety, animal comfort and welfare and its implications on animal productivity and mortality
- Skill training for experts and farmers on proper housing and floor designs, cleaning and disinfection of the farm
- Training for farmers on principles and skills of farm disinfection

Inputs and delivery mechanisms:

- Bedding materials not available in the locality/farm such as saw dust need to be delivered to farmers. Small businesses providing this service need to be supported
- Cleaning requires ample source of water (preferably with pressure) for effectively cleaning the farm premise.
- Farm Hygiene Score Card
- Detergent, disinfectants, antiseptics and protective clothing (overall, boots, head cover, gl oves) are required. The delivery of these logistics is based on the public service delivery system, the veterinary/animal health posts, and private vet clinics in the area.
- Antiseptics: Formalin (10%), Virocyl, Bleach, Savalon, Alcohol, detergent,
- Materials/equipment: protective clothing, sprayer, gloves , brushes

The delivery of these logistics is based on the public service delivery system, the veterinary/animal health posts, and private vet clinics in the area.

Action 1: Provision of adequate space and appropriate flooring system

Note:

- Proper housing design and adequate space for each animal reduces discomfort and disease risk that would result from the accumulation of animal waste. Congestion of animals in small space forces animals to lie on waste and come in direct contact with disease agents.
- Improper flooring such as floors with crevices provide favorable condition for disease agents to multiply and is difficult to clean.
- Improper flooring such as rough floors with wet/muddy and untidy soil floors is a source of discomfort for both the dam and her young and result in the dam not caring for her young
- . Improperly constructed paved floors with sharp edges would also injure the young. Calves that are kept together with adults on untidy and rough floor are likely to pick microorganisms that eventually lead to sickness and death

- It is therefore important to design proper housing and flooring and provide bedding to reduce calf mortality.

Do the following:

- Create awareness and initiate farmers to construct or modify the design of their barns following proper barn and floor designs. For intensively managed dairy farms in peri-urban and urban areas: Mature milking cows need 1.80mX1.10m space, while heifers and calves need 1.7mX1.0m and 1.5mX1.0mX1m, respectively.
- The barn should be designed to be convenient for working, milking, feeding and ease of cleaning.
- Construct one or two pens for small and medium scale farms and keep the calves in a separate pen for up to 4 weeks
- The floor of the calf pen should be at least 20cm higher from the ground. Proper drainage system should be constructed.
- Construct a slanted floor (3-5 degree slope) in the main house suitable for proper drainage and ease of cleaning. Floors should, as much as possible, be constructed from durable material
- Use appropriate slatted floor system for calves (See Fig. 1)
- Provide adequate soft bedding material such as cow mat, straw, sand or sawdust where available for reasonable comfort of the calves and cows.

Don't do the following:

- ☒ Don't allow crevices on paved or soil floors
- ☒ Don't use unleveled and harmful (too slippery/ too rough)floor
- ☒ Avoid using bedding material that is moldy
- ☒ Do not continually rear calves in pens, dirty yards or small paddocks that become heavily contaminated.

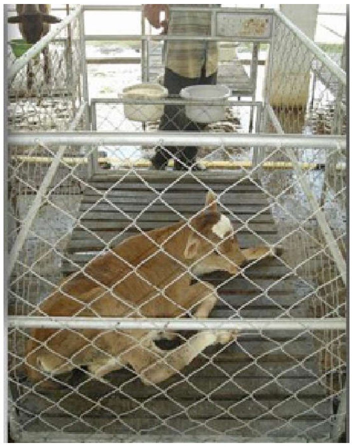

Slatted floor in a calf pen

Calves that are kept together with adults on untidy and rough floor are likely to pick microorganisms that eventually lead to sickness and death

Action 2: Maintaining a clean premise and disinfection

Note:

- Maintaining a hygienic premise is mandatory to protect the calves and the cows from diseases
- Such an environment is also safe for personnel working in the animal yard.
- Standard farm cleaning practice must be formulated for each dairy production system and adhered to ensure a clean environment for maximizing the survival of calves.

Do the following:

- Construct a manure pit and a waste disposal pit for proper handling of manure and other biological wastes. The pit should be deep enough to avoid access by roaming dogs or scavenger birds. Burn the waste while in the pit
- Thoroughly clean the manure at least once a day for concrete floor or once in a month for slatted/bedded/matted floor using locally available broom. The dung and used bedding material should be completely removed during cleaning.
- Thoroughly wash the floor with pressurized water (if paved) at least once a day and drive out all the stagnant slurry in the drainage system

- Thoroughly spray the floor with commercially available and environmentally safe disinfectants such as bleach or Virocyl at least once in three months
- Read carefully instruction provided by manufacturer and/or advise from an animal health expert for dilution, application and precautions during application of disinfectants
- Disinfection could be done by service providers rather than farmers themselves. The service could be provided by the kebele health post and private veterinary clinics
- There must be a farm biosecurity certification system in place. The certification will be granted to the farm by the responsible office as evidence of meeting biosafety and standard hygiene after onsite evaluation or assessment

Don't do the following:

- ☒ Don't dry dust/sweep the floor! Dust will hang in the air and later settle down.
- ☒ Don't allow accumulation of slurry for longer than 2 days
- ☒ Don't directly spray disinfectant chemicals on animals, feeds, water, feeding and watering troughs
- ☒ Avoid the use of dirty water in cleaning the sheds since dirty water can further contaminate the premises
- ☒ Prevent the growth of algae in the water troughs by frequently brushing and cleaning
- ☒ Never put fresh fodder supply over the previous day's left over fodder in the manger. Molds can easily develop on moist forage

Action 3: Using a Farm Hygiene Score Card

Note:

- Monitoring farm cleanliness gives the chance to take appropriate hygienic measure without delay. This will prevent the establishment of diseases causing agents and the risk for disease

Do the followings:

- ✓ Prepare a printed farm hygiene score card (poster) and keep with farm recording book
- ✓ Score your farm using a Farm Hygiene Score Card at least once every month .
- ✓ At least 25% of the animals have to be evaluated and the average should be computed. Score 1 - 2 are acceptable however; if the average is within the poor score range (3 or 4) appropriate measures for to correcting hygienic status of the farm has to be taken.

<table>
<tr><td rowspan="4">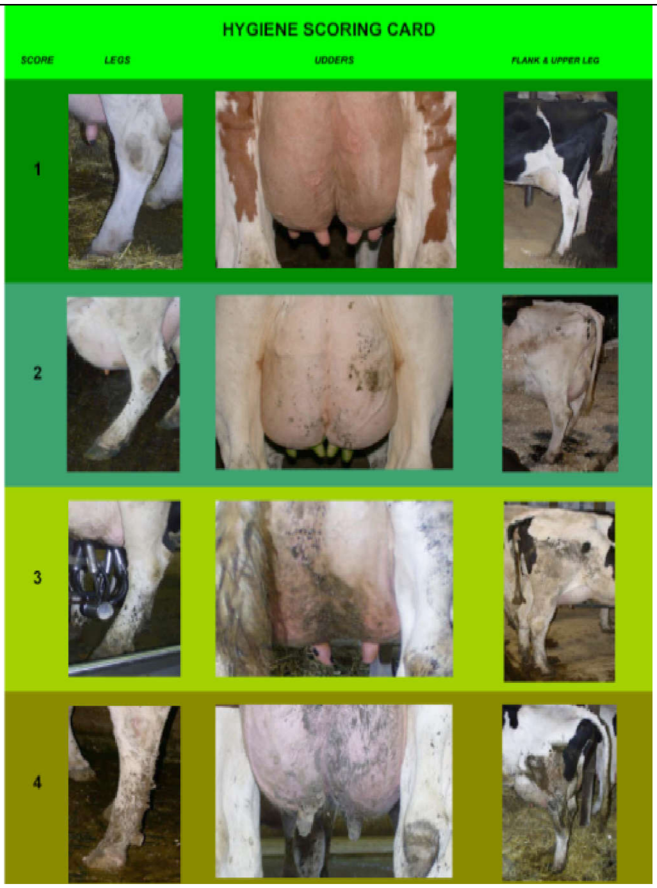

Farm Hygiene Score Card</td><td>Clean lower legs, clean udder and clean flank and upper leg</td></tr>
<tr><td>Relatively clean lower leg with minor dirt bruise, minor tinges on the udder, relatively clean flank and upper leg with minor tinges on the skin around joints</td></tr>
<tr><td>Dry fecal material in the lower leg, Dirty udder with tinges and some fecal material occasionally involving the teats Slightly dirty flank and upper leg region</td></tr>
<tr><td>Very dirty lower leg with fecal material still clinging on the hair, dirty udder/teat with more fecal material, dirty flank and upper leg</td></tr>
</table>

Action 4: Using foot bath/disinfectants at the entry points to the farm

Note:

- Unmonitored entry of new subjects (human or animal) to the farm must be restricted to prevent the entrance and spread of diseases causing agents. Follow the instruction below to ensure its proper implementation

Do the followings

- Put foot bath (at least 30 cm diameter) at the entrance gate or door of the farm (Fig A). Fill the container with antiseptic from veterinary drug store (eg Virocyl at a rate of 5 ml in 2 L of water) or bleach (at a rate of 100 ml in 2 L of water) or caustic soda (at a rate of 10 gm in 100 ml of water).
- Spread straw or put a piece of sack on the foot bath to prevent wastage of the antiseptic
- Ensure foot bathing by every person entering the farm
- If the farm is big and allows entrance of a vehicle, a wheel bath is mandatory (Fig B)
- Change/renew the disinfectant once a week
- Spread calcium carbonate at the entrance of an open shed area to disinfect the shoes of staffs working in the farm
- Additionally, construct hoof dip filled with $CuSO_4$ on the way from the dairy shed to the milking shed for hoof care in large commercial farms
- Do not allow entry of workers of other farms
- Do not enter farm without protective/working cloth and without bathing foot
- Do not take home working /protective wears

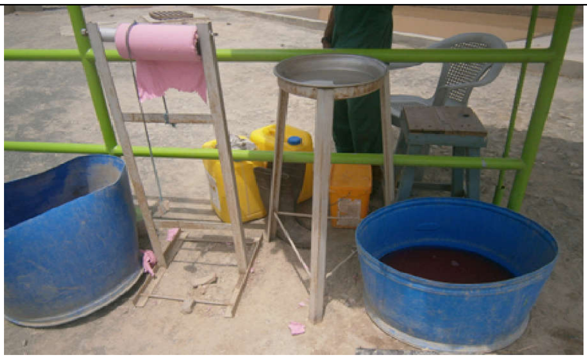

Fig A: Foot bath, hand wash and towel

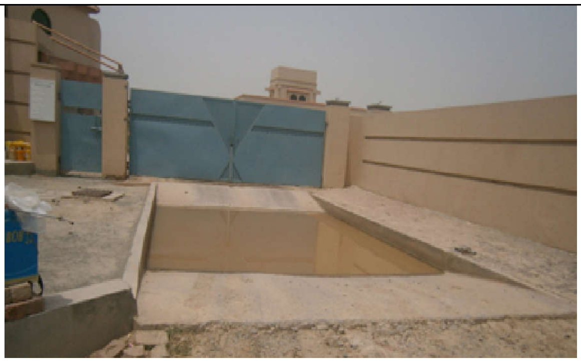

Fig B: Car wheel bath at the main entrance gate

Action 5: Use of protective clothes

Note:

- Note that there are a number of zoonotic diseases (livestock diseases that can be transmitted to humans and cause illness)
- All personnel working in the dairy farm should wear protective clothing dedicated for the purpose of working in the farm.

Do the following:

- To prevent introduction of diseases to the farm, strictly use/dedicate clean working wears (overall, boots, head cover, gloves) for working in the farm.
- Do not enter farm with ordinary cloth. Do not allow entrance of people who visited other farms.
- Warning signs like the pictures below would help alert personnel and visitors. Put such signs at the farm agte.

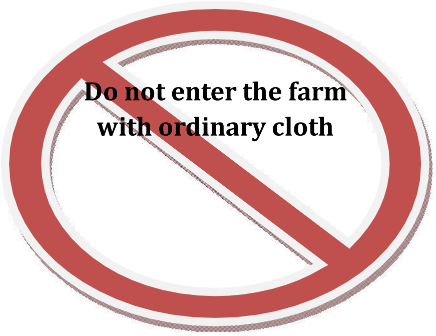

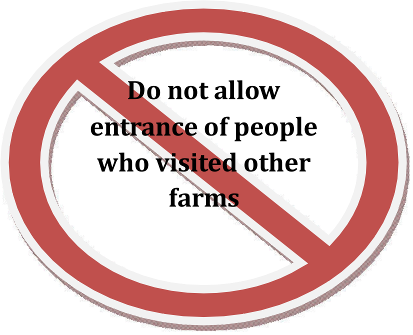

Action 6: Assurance of implementation

- Training (possibly by extension agents) on methods of farm cleaning, and disinfection, using appropriate flooring system.
- Maintenance of standard Farm hygiene (a follow-up of the farm cleanliness, regular floor disinfection and implementation)
- Putting farm biosecurity certification system in place
- The farm hygiene inspection might involve the registration of approved dairy production premises (dairy farm), inspection of milking premises, farm equipment and milk-producing animals, and enforcing satisfactory standards.
- The inspectors also undertake sampling of raw milk and other relevant samples for further analysis before granting the certificate.
- Overall monitoring of activities by Woreda experts is also required.

Pre-requisites: The implementation of this package has certain requirements that need to be fulfilled for its successful implementation. The following are the prerequisites:

- To ensure proper farm cleanliness and bio-safety, there should be a farm biosecurity certification system in place and supervisors should be trained on how to implement the certification
- To grant the certification, there should be regular farm hygiene inspection which involves the registration of approved dairy production premises (dairy farm), inspection of milking premises, farm equipment and milk-producing animals, and enforcing satisfactory standards. The inspectors also undertake sampling of raw milk and other relevant samples for further analysis before granting the certificate.
- Accessible disinfection service. The kebele health posts and private service providers need to be prepared to provide the service,
- Farmers need to be skilled in standard farm cleaning, disinfection
- Farmers need to have awareness and knowledgeable of appropriate housing and flooring system, maintenance of standard Farm hygiene practices
- Overall monitoring of activities by Woreda experts is also required

INTERVENTION 2: PRENATAL CARE OF THE DAM

Description of the intervention: the objective of this intervention is to give the necessary care for the pregnant dam during the last trimester and at parturition. The intervention involves supplying appropriate levels of nutrients (energy, protein, Ca, Mg, Vit A) during the last trimester to ensure a healthy and strong dam that is ready for parturition; separating the dam approaching parturition from the rest of the herd and providing a clean and dry area/house.

Skill and capacity building: Skill focused training is required for EAs and farmers on the preparation of Urea-Molasses Blocks, Multi-nutrient blocks, Urea treatment of crop residues, and production of improved forages that can be used to feed the dam approaching parturition

Inputs and delivery mechanisms: Materials needed to produce the various feed supplements. Urea-Molasses lick and urea treated residue can be introduced as a business initiative (with credit availability, tax relief, provision of land for the business etc). Alternatively, potential cooperative initiatives can also be applied for production at reduced cost. Production of targeted marketable mineral licks (Limestone, local salt licks) can also be another option.

Pre-requisites: Accessibility of molasses, regular monitoring system for implementation and sustainability (EAs will be primarily responsible for monitoring the implementation and should be exempt from other Extension work). Intervention enforcement mechanism must be set in placeto achieve maximum success

Action 1: Feeding the dam during the last trimester (after six months of pregnancy)

Note:

- ☞ Management decisions made during the last trimester of pregnancy are critical and can influence success at calving, survival of the newborn and profitability of the farm.
- ☞ Underfeeding cows prior to calving could increase calf scours and mortality and reduce calf survival
- ☞ Ideally, most cows should enter the calving season with a body condition score (BCS) of 5 (on a 1 to 9 scale??). If the cows are thinner than desired, it is advisable to alter the plane of nutrition to add body condition prior to calving.

Do the following:

The following points are worth noting during prenatal feed management of the dam

- Feed good quality forage and supplement concentrate based on the following schedule depending on the body condition of the cow:
    - ✓ Week 12-7 before calving : Supplement Forage legumes (e.g. Alfalfa, Sesbania, Leucaena), Mix one portion of the forage legume to two parts of dry roughage/grazing free choice or feed Urea-Molasses lick during morning and in the evening with ample amount of water.
    - ✓ Start supplementing 0.5 kg concentrate during week 6 before calving and increase the allowance by 0.5 kg every week to attain a supplementation of 4 kg on the final week before delivery. Local feeds like “Atela” could be used
    - ✓ Stop offering mineral supplements between 14 and 7 days before calving to stimulate the body’s mechanism for drawing on calcium stores in the bones which can help avoid health problems in early lactation (milk fever). Resume feeding minerals for the remaining one week before calving is due.
- Wilt the forage by leaving it out in the sun during the day and preferably chop to encourage the cows to eat.
- Feed the cows in the evening and during the day to optimize intake especially in warm areas.

- Use reference manual for urea treatment of crop residues, preparation of Urea-Molasses Blocks and production of improved forages(Various ESGPIP technical bulletins)

Action 2: Timing of feeding (Konefal's Method)

Note:

- Konefal's Calving Method is feeding the cow at fixed time to promote day time calving.
- The time you feed the cow impact when calves are born.
- In general, cows fed at night tend to calve during the daylight hours (when you have an opportunity to watch them more closely and provide assistance if necessary).

Do the followings:

- Feed cows twice daily, once in the morning (at 11 am. - 12 noon) and again in the evening (at 9:30 to 10 p.m.)
- Start this practice about one month before the first calf is born. Feeding cows using this regime will result in 80% of the cows calving during the day time.

Action 3: Separating the dams around the time of parturition

Note:

- The peri-parturient period is one of the most important times of the reproductive life of the cow. Hence maximum care should be given to the cow approaching parturition.

Do the followings:

- Separate the pregnant animal at least one week before expected date of parturition and for another one week after parturition
- Provide a separate clean and dry area/house for the pregnant cow and newborn
- Provide plenty of fresh clean water immediately after delivery,

- Supplement with the recommended supplements while the animal is in the assigned shelter
- Continue supplementation for the dam during the pre-weaning period to support proper lactation

INTERVENTION 3: NEONATAL CARE OF THE CAL VES

Description of the intervention: the objective of this intervention is to give the necessary care to newborn animals from birth up to 1 months of age. The neonatal care includes induction of breathing through clearing the air way and chest massage, improving peripheral circulation, navel treatment, feeding colostrum, and measuring birth weight that helps determine whether the calf needs further attention.

Skill and knowledge capacity building: Training on neonatal care of calves, management and care of newborns to EAs and farmers

- Training on neonatal care of calves, management and care of newborns to kebele experts and farmers
- Skill training on induction of breathing for kebele experts and farmers
- Skill training on navel treatment for kebele experts and farmers
- Skill training on calf body condition scoring for kebele experts and farmers
- Skill training on assessment of sick newborn calves for kebele experts and farmers

Inputs and delivery mechanisms:

- Equipment and other materials needed to provide neonatal care include Plastic bulb pipette (needed to clear the nasal cavity)**,**
- Calf box with comfortable and warm bedding, Warm cloth to wrap the calf, Milk feeder, colostrum replacement or Foster colostrum from dams that have recently delivered in case of dead dam or poor mothering,

- Detergent, Antiseptics, Clean thread and blade, weighing scale, and Protective wear for professionals (as deemed necessary) are all required
- The inputs could be provided by the kebele health post, private clinics/drug stores if available in the kebele. If private service providers are not available, new businesses could be initiated (which will also be a job opportunity for the kebele youth) with the support of the project, credit providers and kebele administration.

Pre-requisites: Accessible disinfection service, Regular monitoring system for sustainability of implementation (EAs will primarily b eresponsible for monitoring the intervention), and Enforcement mechanism put in place are some of the pre-requisites for successful implementation.

Action 1: Induction of breathing

Note:

- Survival of the newborn is dependent on the rapid onset of normal, spontaneous respiration.
- In a normal calf if parturition occurs normally, respiration usually commences after 30 seconds of birth, being irregular at first before settling down to 45–60 breaths per minute.
- Once birth is complete, it is important first to ensure that the upper respiratory tract is cleared of fluid, mucus and attached fetal membrane. This can be done with the aid of fingers or, preferably, with a simple suctio n device (plastic bulb pipette)

Do the following:

- ✓ Start assisting the dam and the calf after washing your hand or use protective gloves at all times
- ✓ Clean the nostrils from any mucus or fluid by gently squeezing the muzzle
- ✓ Hold the newborn by the hind legs and lift upside down for a few seconds to expel fluid from the lungs (as shown in Fig. 4)
- ✓ Insert a finger into the mouth, or gently pull out the tongue until the newborn produces sound

✓ Brisk rubbing or gentle massage of the chest with towels frequently provides the necessary tactile stimulus to stimulate respiration.

✓ Provide extra care for premature, weak and small calves or seek help of an animal health professional

☒ Don't insert sharp and dirty material into the nostril to induce sneezing as this may cause injury

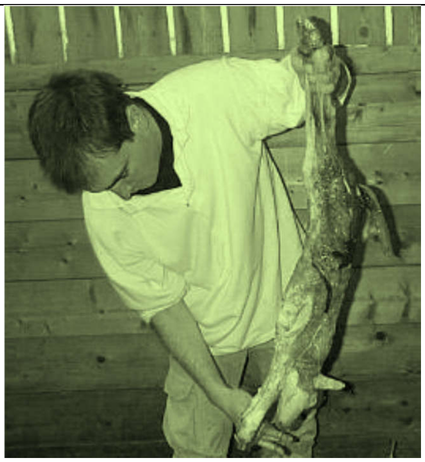

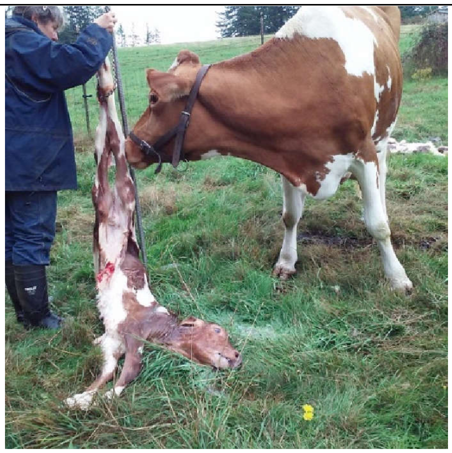

Holding the calf by the hind legs and cleaning the nostril from mucus

Action 2: Navel treatment

Note: The umbilicus usually ruptures passively in cattle. It is hence important to handle it properly.

Do the following:

- Tie the umbilical cord at 5 cm (approximately three fingers) length with clean string or thread (Fig. 5a)
- Cut the remaining part of the umbilical cord and dip or rub the stump with antiseptics (iodine tincture or savlon) (Fig. 5b) or apply antibiotic spray (Fig. 5c)
- Carefully observe the umbilicus to dry and heal over the next few days. If it has not dried and sealed off, immediately seek the help of an animal health professional

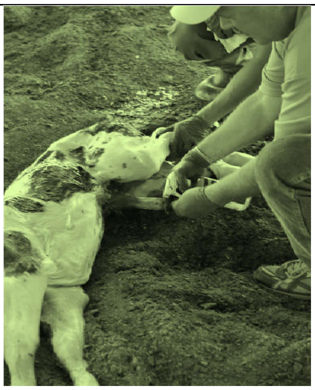	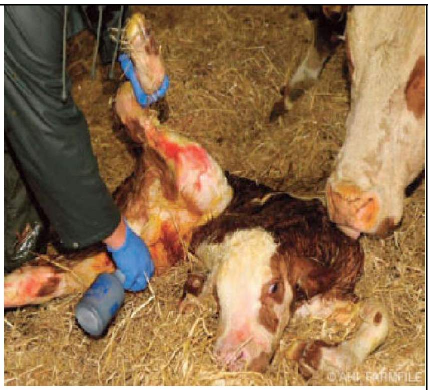	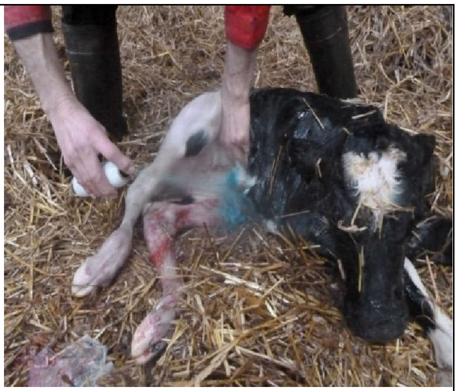
Tie the umblical cord	Dip the navel stump in iodine tincture	Spray the navel stump with antibiotic spray

Pre-requisites: The implementation of this package has certain requirements that need to be fulfilled for its successful implementation. The following are the prerequisites:

- Inputs and services listed under section 5.3.3.3 need to be availed for farmers. The district veterinary clinic and private service providers need to be prepared to provide the service,
- Farmers need to be skilled in standard neonatal care of the calf
- Farmers need to have awareness and knowledgeable of appropriate care and handling of neonates
- Overall monitoring of activities by Woreda experts is also required

Action 3: Measuring/assessing the birth weight, health status and keeping records

Note:

- Measurements taken at birth are very important to make decisions whether the calf requires further assistance.
- Birth weight of a calf is known to affect its survival chance, calves weighing far less than the average birth weight of the breed are highly li kely to die during the neonatal period
- It is important that development agents/veterinary assistants be informed of the average lamb/kid birth weights of the breeds in their locality

Do the following:

- Measure body weight with a weighing scale where availab le
- Subjectively assess the weight/condition category as "Very small", "Normal", or "Large" size where a weighing scale is not available
- Provide extra neonatal care for premature, very small or underweight calves
- Assess the health of the newborn from body reflexes (palpebral reflex: touch the eyelid; anal reflex: touch the anus, or pedal reflex: pinch the interdigital skin) and seek help from animal health professional for sick offspring
- Record all other birth data (date of birth, weight, and condition of birth) on a predesigned format
- ☒ Don't herd or keep weak and small calves with adults other than the dam at any time at least for the first week of life

Don't separate calves from the damat least for the first week of life to strengthen the dam-offspring bond

Action 4: Improving thermoregulation in the calf

Note:

- ☞ Hypothermia (abnormally reduced body temperature) occurs when the energy metabolism is insufficient to maintain body temperature. Following birth, the body temperature of the newborn falls quickly.
- ☞ The newborn has little subcutaneous fat and hence insulation is poor.
- ☞ The body surface is wet and thus heat is lost due to evaporation.
- ☞ It is, therefore, very important to stimulate the calf's peripheral circulation to prevent hypothermia. Hypothermia can be reduced in two ways, by improving feed intake for energy production and by reducing heat loss through insulation.

Do the following:

- Ensure that there is adequate milk intake
- Dry the entire body of the newborn with a clean cloth and/or allow the dam to lick it

- Move the newborn and the dam to a dry and clean place with good bedding
- If the ambient temperature is cold or if the newborn is too weak to stand, provide warmth with brood lamp or coal fire or wrap with piece of blanket or old cloth while in the calf box for about 1 hour. Continue for a while if the calf is still weak
- Don't overheat the newborn or continuously incubate for too long as this might result in desiccation or extreme dryness
- Never pour cold water on the body of the newborn as moisture can aggravate hypothermia

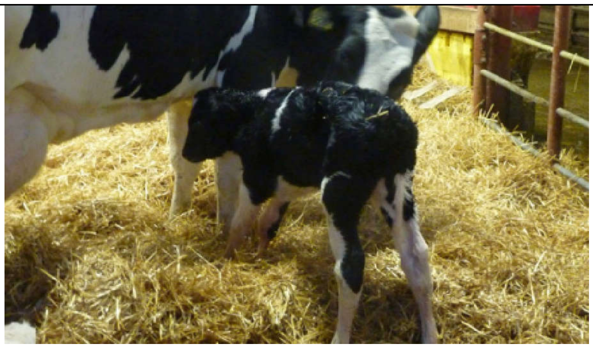

Calf is kept with the dam in a dry, clean area with good straw bedding

Action 5: Feeding colostrum (1-4 days)

Note:

☞ Colostrum is critical to survival of the newborn calf.

☞ The immune system of newborn calves is not completely developed and the immunoglobulins in colostrum contribute a substantial component of the immune protection in newborn calves.

Do the following:

- Check if the dam is producing sufficient colostrum by test-milking all teats
- Make sure the calf suckles the dam immediately after birth or within 6 hours at the latest. Make sure the calf suckles freely for the first day and then three times a day up to day four or bucket feed four to five times a day.

- Calves should receive 5 to 6% of their body weight (1.5 to 1.8 liters for a 30 kilo calf) as colostrum within the first 6 hours and again within the subsequent 6 hours.
- If the calves are not able to nurse or the cow's production of colostrum is insufficient, colostrum from other cows or replacer colostrum supplements may be necessary.
- Ideally, colostrum should be collected from cows within 24 hours of calving and fed fresh. Colostrum can also be collected, frozen and used later.
- When collecting colostrum, consider freezing it in "serving" portions, or about one liter per container. Once the frozen colostrum has been thawed (warmed back) it should not be re-frozen.
- Frozen colostrum should be slowly warmed in warm water to a final temp of 40-45 degrees centigrade. The colostrum should be stirred frequently during thawing.
- Prepare artificial colostrum as a last resortone egg (protein source) + half liter fresh warm water + half liter whole milk (source of lactose and milk protein) + one teaspoonful of oil (energy).
- If the newborn is not suckling for any reason, milk the dam and feed using clean feeding utensils such as nipple feeders, or bucket feeder.
- Calves must be provided with adequate clean drinking water at all times
- Never use animal colostrum for human consumption (avoid human competition with the newborn if the practice is common in the area)
- If colostrum is bottle fed, Don't use a nipple that does not match the calf's ability to swallow. Especially the first feeding of colostrum can cause problems if the feeding rate is faster than swallowing rate.

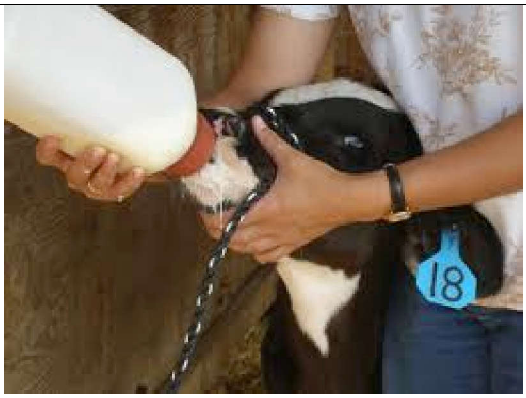<br>Bottle feeding	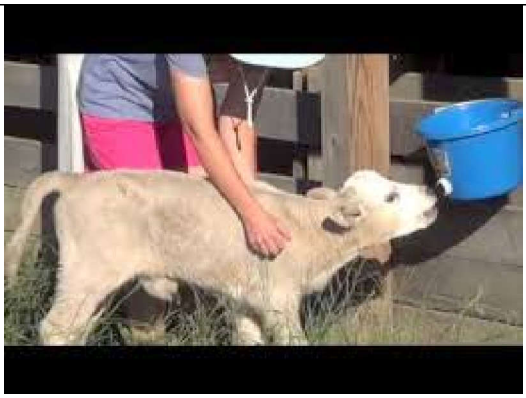<br>Bucket bottle feeding. Put at natural height of the calf for easy suckling

INTERVENTION 4: PRE-WEANING FEEDING MANAGEMENT OF THE CALVES

Description of the intervention: Diseases affecting older calves occurring until weaning can be considered postnatal diseases. The objective of this intervention is to give the necessary nutritional and health care tothe calf during the pre-weaning period from week one to 4 months of age. It includes appropriate milk feeding, preferential and separate feeding (pen feeding).

Skill and knowledge capacity building: skill training is required for EAs and farmers on construction of creep feeders, and method of preferential feeding.

Inputs and delivery mechanisms: The following inputs are necessary for proper implementation of the intervention: Feeding management, feed ingredients, and construction materials for creep feeder. Intensively use posters, slogans for awareness creation.

Pre-requisites: Accessibility of supplementary feeds, Regular monitoring system for implementation sustainability (EAs will be primarily responsible for monitoring the

implementation and those assigned to this work are expected to be exempted from other assignments to concentrate on this work.)

Action 1: Appropriate milk feeding

Note:

- ☞ Milk contains all the necessary elements to meet the demand by the newborn calf. It is thus essential that the newborn consumes adequate milk during this period.

Do the following:

- Check if the dam is producing sufficient milk by test-milking both teats
- Make sure the calf suckles properly. Calves should be fed milk at approximately 10% of their body weight. If handfed, milk can be mixed with other dairy products (whey or skim milk) at this stage and should be fed at body temperature.
- If the dam does not produce enough milk or if it dies at calving, milk from another cow can be fed using nipples or bucket feeding.
- Feed the total amount of milk allowance at 3 or 4 equal intervals. The amount of milk can be gradually cut down by feeding increasing quantities of calf starter and lush forage. Follow the following feeding guide for each calf:
    - 4 days-4 weeks: 2.5 kg milk, 50 g calf starter and free choice green grass;
    - 4 -6 weeks: 3.0 kg milk, 50-100 g calf starter and free choice green grass;
    - 6-8 weeks: 2.5 kg milk, 100-250 g calf starter and free choice green grass;
    - 8-10 weeks: 2.0 kg, 250-350 g calf starter and free choice green grass;
    - 10-12 weeks: 1.5 kg, 350-500 g calf starter and free choice green grass;
    - 12-16 weeks: (Weaning) 500-750g calf starter and free choice green grass.
    - Give the calf its milk immediately after milking when the milk is at body temperature to avoid digestive troubles.
- Fix a poster containing the information/guideline above on the wall of the farm for easy reference
- Train the calves to feed from buckets and start eating calf starters

- To train the calf to consume calf starter concentrate, rub a small amount of the starter feed on to the calf's mouth after milk feeding for a few days after which the calf will be accustomed to it.
- To teach the calf to consume from a bucket, put two fingers in the calf's mouth so it starts to suck, gently lower its mouth into the colostrum/milk in the bucket, slowly remove your fingers and the calf will start drinking from the bucket.
- Control the bucket feeding of milk as greedy calves can swallow large quantities of milk some of which may end up in the windpipe leading to pneumonia.
- Feed the calf only milk up to 3 weeks of age
    - Place hay, water and protein supplement (such as high quality lush forage) near the calf to encourage early eating and drinking starting from 3 weeks of age. This helps development of the rumen.
- Observe that the composition of starter feed supplements fulfils the following:
    - *Calf starter:* Calf starter is a highly nutritious concentrate mixture formulated to contain all the nutrients in proper proportion required for optimum growth and is used as a partial substitute for whole milk in the ration of calves. The starter should contain 18% crude protein, low fibre, 0.7% calcium, 0.45% phosphorus and vitamins A, D and E as the calf is consuming little milk and its rumen is still not fully developed. Since quality of protein is very important to calves until their rumen is fully functional, it is good to include animal protein supplements such as fish meal in calf starters. Urea should never be included in calf starters. The following table presents a simple calf starter formula.

Example of a simple, acceptable calf starter.

Ingredient	% of total ration
Corn, cracked	52.0
Oats	20.0
Soybean meal	20.0
Molasses	5.0

Limestone	1.0
Dicalcium phosphate	0.25
Salt, trace mineral	0.20
Animal fat	1.50
Vitamin supplement	0.05 (or to provide needed vitamins)
Other (coccidiostat, buffer)	as needed

- *Roughage:* only high-quality forages supplemented with concentrates (calf starter) early in life should be offered. If hay is used, it should be of high quality, fine texture, mixed with legumes and fed *ad libitum*. If calves are on pasture, it is best to always graze them ahead of adults to minimize parasite infestation. Common roughages offered to calves include sweet potato vines and freshly harvested, wilted legume forages like Alfalfa and similar high quality forages.
- *Water:* Calves should be offered fresh water *ad libitum* in addition to milk. It is estimated that efficient feed conversion requires about 4 kg of water for each kilogram of dry feed the calves eat.

- You can use different calf feeding methods as appropriate. The following are the most common methods:
  - ***Individual suckling:*** The natural way for a calf to feed is to suckle the dam after she is milked. Do not completely milk the cow out or there will be no milk left for the calf. A simple way to feed the calf is to leave one quarter unmilked and allow the calf to suckle this quarter after each milking. Leave the calf with the dam for around two hours and then remove it until after the next milking.
  - ***Foster mother or multiple suckling:*** One milking cow may be assigned a number of calves to suckle, depending on the level of milk production. This is practical only on farms with several cows lactating at the same time.

- ***Nipple suckling:*** A rubber nipple is fixed on a milk bottle and the calf is trained on how to suckle. The alternative is to put milk into a bucket and insert a flexible plastic hose pipe with one end attached to a nipple.
- ***Bucket feeding:*** The most commonly used method is bucket feeding. The calf is trained to drink milk from a bucket. Stainless steel buckets, where available, should be used for hygienic reasons as plastic buckets are difficult to clean.

- Wean the calves on high-quality pasture and/or fodder to maintain high growth rate.
  - A concentrate mixture of (e.g. 35% oats, 5% linseed cake, 30% bran, 10% corn, 20% groundnut cake) may be fed to the calves.
  - It is good to hang a mineral lick where the calf can get the minerals it needs.
- Don't feed milk from clinically mastitis cows
- Don't feed excess milk to the calf, it can cause scouring. If scouring occurs, reduce the amount of milk by half and mix the remainder with an equal amount of water.

Action 2: Preferential and separate feeding

Note:

☞ As the calf grows, its nutrient requirement also grows. However, it can be difficult to reach a maintenance feeding level when there are competitors.

☞ The calf requires a separate extra feeding care that can be provided as follows

Do the following:

- Use only freshly prepared feed daily
- Observe strict cleanliness and hygiene to prevent scouring (use clean milk feeders and other feeding and watering equipment)
- Don't over feed concentrate
- Don't feed moldy feed

- Don’t give urea treated roughage before four (weaning) preferably 6 months of age.

INTERVENTION 5: REPRODUCTIVE MANAGEMENT

Description of the intervention: Dairy farms may encounter up to 15% stillbirths and neonatal mortality. Cows that start breeding at an early age are likely to have a longer reproductive life. However, disorders like dystocia can impair reproductive success and survival of their newborns. The objective of this intervention is to manage reproduction in the cow to reduce neonatal mortality while improving reproductive performance of the cow.

One method of reproductive management is introducing controlled mating, and estrus synchronization

Skill and knowledge capacity building: Skill training and awareness creation on cattle controlled breeding to EAs and farmers. Training and Demonstration on the application of controlled mating

Inputs and delivery mechanisms: Input for estrus synchronization (e.g. hormones, PRID, CIDR, syringes, rectal and examination gloves), pelvimeter for measuring the pelvic area, Dystocia kit, Pregnancy Diagnosis kit (ovucheck rapid, Preg-tone).
Some of the items can only be procured from foreign market hence require import through dealers.

Pre-requisites: Sustainable and feasible estrus synchronization scheme. Availability of drugs and equipment used for the protocols and for the PD. Regular monitoring system for implementation sustainability (EAs will be primarily responsible for monitoring the implementation), Support of research centers such as breeders if the opportunity exist.

Action 1: Controlled breeding

Note:

- Cows whose breeding/mating is controlled are much easier to manage.
- Several disorders that render both the cow and its neonate at risk of death can be minimized through breeding management.
- Calves born from heifers with dystocia (difficulty of birth) are more likely to die early.
- Cows that give birth at similar time can be used to foster raise calves whose dams are dead or can't suckle. This requires synchronization of estrus.
- Remember parity of the cow has relation with reproductive disorder that can influence the survival of the calf (mortality decrease with increasing parity that is; calves from cows with more calving experience have higher chance of survival)

Do the followings:

- Prepare a recording system and register all individual animals before deciding for breeding.
- Determine a target Age at First Breeding (which should be 13-15 months) and Age at First Calving (should not be less than 24 months) for breeding heifers in the farm.
- Perform Pelvimetry (Measure the pelvic area) of the breeding heifers to determine the pelvic size (see Figure top panel) and don't breed heifers with very small or very large pelvic measurement.
- Heifers that have very small or very large pelvic size are likely to suffer from dystocia and hence have weaker calves that die early.
- Pelivemetry is performed only by an experienced veterinarian
- Selection of the Bull type for breeding is also important because some bulls tend to give larger calves that often predispose to dystocia,

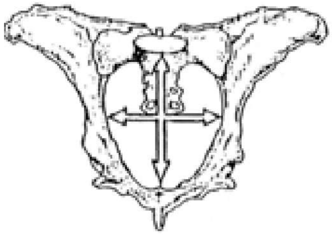

Pelvic area (cm^2) = height (vertical) X width (horizontal) dimensions of the pelvic opening.

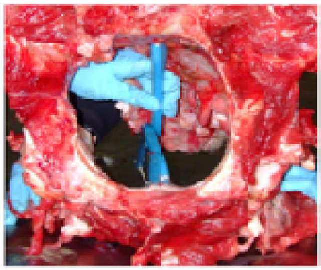

Position of the Rice pelvimeter for measuring pelvic height

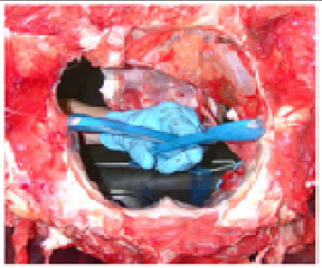

Position of the Rice pelvimeter for measuring pelvic width

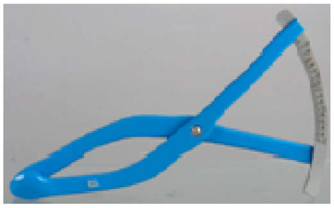

Rice Pelvimeter for measuring pelvic area

A normal 275 kg yearling heifer should have a pelvis at least 11cm wide and 12 cm high (132cm^2 pelvic area) to deliver a 30 kilo calf. Heifers with <10cm wide and or <11cm height dimension should be considered for culling

A heifer 12-14month of age and 270-300kg weight will have pelvic area of 140-180cm^2
A cow 18-19month of age, and 800kg body weight, will have pelvic area of 200-220cm^2

Figure: how to perform pelivemetry in cow/heifer and make decision

- Assistance to a calving cow requires good knowledge of parturition. Knowing when to assist the cow is very important. Either premature or delayed intervention can seriously influence the survival of the calf and subsequent reproductive life of the cow.
- Seek assistance of veterinarian whenever dystocia occur (Follow the guideline given below for calving assistance) (Skill Training on Calving assistance is required)

General guideline for calving assistance

- **The water bag appears in the birth canal and will normally break after 30-60 minutes. It is followed by a period of restlessness and position changes.**
- **The feet appear in the birth canal and delivery is completed after 1hour of hard labor. If the animal has not made any progress after 1-1½ hours, assistance should be given.**
- **Don't wait too long to interfere. Wait only ONE HOUR after the water bag appears**
- **Make sure the three structures are present in the birth canal before pulling (two front feet and a head or two hind feet and a tail). The appearances of one or two structures alone are indications of an abnormal calf presentation**
- **Comfortably restrain the cow before intervention. The best situation is to have a pen with a head catch.**
- **Don't attempt the job without plenty of clean water and detergent or disinfectant**
- **The basic guidelines are *clean and gentle***

3.2 HEALTH RELATED INTERVENTIONS

INTERVENTION 1: PREVENTION AND CONTROL OF DISEASES CAUSING CALF DIARRHEA

Description of the intervention: Acute diarrhea accounts for approximately 75% of the mortality of dairy calves under 3 weeks of age.

The main objective of this intervention is to introduce prevention, control and management strategies to reduce calf mortality due to diarrhea. This requires several steps among which vaccination of calves, vaccination of the cow to boost passive transfer of immunity, Adequate and timely Colostrum feeding, availing isolation and quarantine area, rehydration of scouring calves and seeking help of animal health professionals for treatment of sick calves

Diarrhea is not always the result of infectious agents. It can also be the result of stress, poor management and poor nutrition.

Neonatal diarrhea could be caused by nutritional disorders, viruses or bacteria; digestive upsets leading to scours are generally a major cause of death in young calves.
The most important pathogens associated with calf diarrhea are enterotoxigenic *Escherichia coli*, rotavirus, corona virus, *Cryptosporidia* sp. *Salmonella* sp. and Coccidiosis (*Eimeria* sp.).

- *E. coli* may affect calves as young as 16 to 24 hours of age but it is most common in calves 1-7 days old.
- *Clostridium perfringens* type C is most common in calves 1-3 weeks of age. This bacteria is a soil born organism and causes calf scours on many unhygienic farms.

- Salmonella typhimurium is the most common cause. Calves are usually affected at 1-7 days of age. The source of Salmonella infection in a herd can be from other cattle, birds, cats, rodents, water supply or human carriers.
- Rota virus infection can occur in calves as young as 0-28 days old; however, most outbreaks occur when calves are 1-6 days old
- Coronavirus can occur in calves 0-28 days old but it is most common in calves of 7-10 days of age. The virus is not as resistant to degradation as Rotavirus. Adult cows shed the virus in feces and contaminate calving areas. Rota- and coronavirus are hardy and survive for months in moist and cool conditions
- Bovine viral diarrhea (BVD) virus infection is common in young calves. Diarrhea begins about 26 hrs to 3 days after exposure and may persist for quite a long time
- Cryptosporidium is most common in calves 1-3 weeks of age. As a rule, cryptosporidium is detected in combination with coronavirus, rotavirus, and/or E. coli.
- Coccidiosis is seldom a problem in young calves but occur in calve 1 week of age. However; outbreaks associated with stress, poor sanitation, over-crowding, or sudden changes in feed in calves 3-4 weeks of age and older have been reported.

Skill Capacity building: handling a scouring calf requires knowledge and skill. Awareness creation to farmers and demonstration to EAs/Farmers are useful. LEAs must know the need for colostrums feeding, how to manage sick calves and how to best prevent calf scours

Inputs and delivery mechanisms: The following inputs are required

- Vaccine: bovine E coli vaccine (K99 bacterin), bovine rotavirus vaccine and bovine corona virus vaccine
- Antiparasites: Albendazol, Tetramizol,, Fenbendazol, Decoquinate, Sulfadimidin, Amprolium, Monenzine sodium

- Antibiotics: Penicillin (also as Penstrep), Neomycine, Spectinomycine, , SMZ-TMP (Bacterium) tablets
- Supportive treatments: Oral rehydration salt (ORS), flunixin meglumine (Benamine), Normal bovine plasma, Dextrose saline, normal saline, ringers lactate
- Antiseptics: Formalin (10%), Virocyl, Bleach, Savalon, Alcohol (denatured), Detergents
- Materials/equipment: protective clothing, vaccination and treatment syringe and needle, sprayer, cotton, gauze, gloves

Pre-requisites:

The success of this intervention relies on availability of the inputs and the service delivery system. Therefore, all the inputs listed in the "input and delivery mechanisms" should be available for implementation of the activities listed under each intervention.

Materials could be availed through public (MoLF) or private animal health service delivery clinics/posts

Action 1: Vaccination

Note:

- Vaccination helps the calf to cope with diseases challenges in the new environment.
- Prevention of diarrhea can be made indirectly by vaccinating the pregnant dam or by directly vaccinating the young.
- Vaccinate the dam close to the time of parturition and then timely provide adequate colostrum to the calf to give protection for few months until the calf itself is able to produce antibodies.
- The age at which vaccination is made for the calf may vary for different diseases. Usually colostrum must be fed within few hours of birth while vaccination of calves is made after eight weeks of age.

Do the followings:

- Vaccinate the breeding female before pregnancy to strengthen passive transfer of immunity through the colostrums right after delivery
- Follow the instructions by the manufacturer for vaccinations
- Don't vaccinate pregnant cows/heifers at early stage of pregnancy and calves before six months of age
- Ensure passive immunity through adequate and timely provision of colostrums (***refer to the SOP for colostrum feeding in this manual***)
- Vaccinate all calves above 8 weeks of age for bovine *E coli* vaccine (K99 bacterin), bovine rotavirus vaccine, corona virus, and BVD vaccine
- Vaccinate the pregnant female around one month before parturition with bovine E coli vaccine (K99 bacterin), bovine rotavirus, corona virus vaccine, and BVD vaccine to strengthen passive transfer of immunity through colostrum
- Preferably use 2ml, IM injection (side of the neck) of ***Rotavec® Corona*** vaccine that can protect against Rota, Corona and E coli
- Follow the instruction by the manufacturer for vaccination and withdrawal

Generally, the following steps might apply for vaccination

- Read the labels on the vaccine vial every time you are about to vaccinate even if you know the label by heart
- Record in a notebook which calf you are vaccinating, the date and the vaccine batch to avoid mistakes of revaccination
- Use the proper syringe and a needle (18 gauge and at least 2 cm long)
- Disinfect the needle between vaccination if you are not using disposable needles
- Inject the needle on either side of the neck, pinch the skin and inject 2 ml vaccine parallel to the skin (SQ) and repeat as prescribed for the vaccine
- Make sure you store the vaccines properly usually refrigerated at +4 degrees
- Unused vaccine must be discarded. Partially used vaccines will be contaminated and are dangerous for the animal if used

- If you inject yourself mistakenly, seek help of a physician immediately
- Don't inject needle in dirty area otherwise it might become infected
- It is good to replace the vaccination needle once for every 20 animals
- Don't vaccinate pregnant cows/heifers in the first trimester (first 3 months of pregnancy)
- Don't vaccinate unhealthy and wet animals
- Don't wipe injection sites with disinfectant, this can reduce the effectiveness of the vaccine
- Don't give other medication at the time of vaccination

Action 2: Isolation and quarantine

Note:

- Remember some diarrhea causing agents could be shed to the environment even before clinical symptoms are apparent and during convalescence. This can contaminate the environment and hence other susceptible animals.
- Separating the sick calf helps to give sufficient attention for care and prevention of the spread of the diseases.
- Close observation of calves at feeding to identify scouring animals as early as possible for remedial treatment will prevent dehydration and secondary disease leading to chronic ill-thrift and mortality.

Do the followings:

- Avail a separate, dry and clean barn for isolating sick animals
- Newly introduced animals (purchased or returned from market) should also be separated for one week
- Quarantined sick animals must be fed and watered using a separate trough and kept until the illness has gone
- Provide proper care (good feed and water; dry and warm bedding and well ventilated) and seek help of animal health professionals for treatment/management of isolated animals

- Clean (remove the feces with brush/spade and wash with detergent water) and disinfect the barn using bleach or 10% formalin if the floor is contaminated with feces
- Be sure equipment, boots and hands are thoroughly cleaned after handling sick animals
- Don't mix sick and apparently healthy animals in the isolation barn
- Don't delay to seek help of animal health professionals for cases of diarrhea

Action 3: Rehydration of diarrheic calves

Note:

- o It is essential to replace lost fluid due to diarrhea as a first measure to save the life of the calf.
- o The critical to remember that dehydrated calves need 6.5 to 8 liters of fluid daily to correct the dehydration. They also need an adequate caloric intake for maintenance and growth

Do the following during rehydration of the calf:

- ✓ Provide oral rehydration salts (readymade/home prepared) immediately upon detection of diarrhea (10% of BW three times/day) until diarrhea stops
- ✓ Prepare homemade rehydration solution as follows
  - ✓ **Solution 1**
    - 6 tablespoonful of sugar
    - ¼ tablespoonful of salt
    - Mix into 1 liter of clean water and use 7 or 8 liters every 24 hours

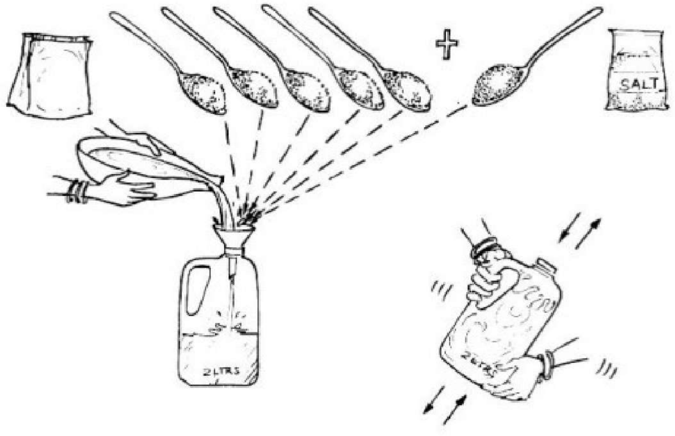

- ✓ **Solution 2**
  - ¼ tablespoonful of salt
  - ¼ tablespoonful of baking soda (optional)
  - 6 tablespoonful of sugar
  - 2 tablespoonful of lemon juice (optional)
  - Mix into 1 liter of clean water and use 7 or 8 liters every 24 hours
- ✓ Rest diarrheic calf until recovery
- ✓ Don't stop provision of ORS before diarrhea stops
- ✓ Oral rehydration salt (ORS) is a supportive treatment hence do n't consider ORS as a final and curative treatment

- Avoid feeding milk for 1-2 feeds. Give fresh water, concentrates and forage.
- Don't stop provision of ORS before diarrhea stops

DIARRHEA: NUMBER ONE KILLER OF DAIRY CALVES!!

- Don’t give antibiotics to treat scours resulting from over-feeding or digestive upsets. Blood scours (mostly caused by coccidian or other microorganisms) require veterinary treatment and management changes to improve hygiene.

Action 4: Prevention and Treatment of sick animals

Note:

- Before treating a scoring calf, it is essential to determine why it is scouring. Take the calf's temperature using a rectal thermometer. If body temperature is above the normal range (38.5-39°C), fever medications such as Flunixin meglumine and antibiotics must be given.

Do the followings:

- ✓ Employ the necessary standard hygienic and biosecurity measure to ensure prevention of diseases from entering the farm
- ✓ Check rectal temperature of the sick calf and if there is fever; animal health professional must treat the calf with the following medications
    - Give fever medication – flunixin meglumine (1 mg/kg IV or IM every 12-24 hrs)
    - Give diarrhea medication – Kaopectate or Pepto-Bismol
    - If failure of passive transfer of immunity (inadequate colostrums) is suspected, give normal bovine plasma 20-80 ml/kg
    - Rehydrate the animal with ORS (homemade or commercial)
    - If bacterial infection (collibacillosis, salmonellosis, or clostridium) are suspected give parenteral administration of ceftiofur (2.2 mg/kg IM or SC every 12h) and amoxicillin or ampicillin (10 mg/kg IM every 12h)--or sulfonamides (25 mg/kg IV or IM every 24h) for treating calves with diarrhea and systemic illness. If there is no systemic illness, no antibiotic treatment is needed
    - If protozoan infection (coccidiosis, Cryptosporidium) is suspected, give either of Decoquinte, Monenzin sodium, Amprolium, Sulfa-antibitics, or SMZ-TMP tablet based on the tentative diagnosis

- If viral (rotavirus, corona, BVD virus) infection is suspected, give only supportive fluid, provide good food and rest the sick calf

✓ Don't treat sick calves yourself if you are not an animal health professional

✓ Don't use antibiotics and anthelmintics purchased from uncertified sources (forged drugs)

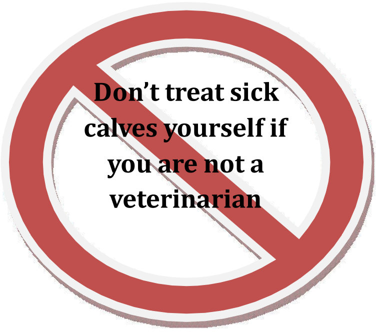

Action 5: Laboratory Confirmation of diseases causing agents

Note:

- Appropriate samples for specific identification must be taken by veterinary health expert. A veterinarian caring for the sick individuals must always take samples from sick animals for the purpose of confirmatory diagnosis
- Samples must be properly labeled, kept in appropriate preservative or maintained in the proper cold chain until they reach laboratory.
- Samples should be stored locally only temporary until they are transported to the laboratory

Do the followings

✓ Collect at least 10 ml of blood from jugular vein (alternatively from tail vein underneath the tail) in the neck region using a vaccutainer tube (fill 2/3 of the tube). Clean the skin at the site of penetration using alcohol/savalon swab

✓ Always label each sample (date and identification code). Keep the tube slightly tilted at room temperature overnight (or for at least 2 hrs) to harvest serum if this is the purpose

- ✓ Serum samples should be decanted into a cryovial, labeled and kept in deep freezer at -21 ^{0}C until analysis
- ✓ Fecal samples should be directly taken from the rectum using glove and kept in a universal container. Depending on the protocol, a preservative can be used for the feces
- ✓ Follow instructions of the laboratory technologist for taking, storing and transporting other samples (please refer to SOP for laboratory procedures in identification of diseases causing agents)
- ✓ Nasal swabs must be taken aseptically in a container designed for this.
- ✓ Skin scrapings should be taken using a scalpel blade right at the periphery of the lesion (not from the middle) scrapped until a healthy tissue is visible. Place skin scrapings in appropriate preservative as per the instruction of the laboratory technologist.
- ✓ Samples that require exporting, in case of absence of proper laboratory facility locally, should be temporarily stored at the National Animal Health and Diagnostic Center at Sebeta

INTERVENTION 2: PREVENTION AND CONTROL OF DISEASES CAUSING PNEUMONIA IN CALVES

Description of the intervention:

Pneumonia occurs primarily in housed calves over 2 months of age. It is caused by infection with respiratory viruses and inadequate ventilation and may accounts for about 15% of calf mortality from birth to 6 months of age. Clinical disease can usually be avoided if proper attention is paid to overcrowding, inadequate ventilation and air movement and mixing of age groups. Calves that have suffered from scour are more likely to develop pneumonia later in life. The objective of this intervention is to introduce prevention, control and management actions to reduce calf mortality due to pneumonia.

Skill Capacity building: Awareness creation on causes and risk factors of pneumonia and training for the livestock extension agent (EA)/Farmers on husbandry skills, vaccination, colostrum feeding, management of sick animals, treatment and prevention methods.

Inputs and delivery mechanisms: The health post need to be strengthened with man power, equipment and supplies. The following list of materials and supplies are required.

- Vaccine: IBR vaccine, Pasteurella vaccine, Manheima vaccine
- Supportive medications: Flunixin meglumine (Banamine); ringers lactate
- Antibiotics: Penicilline, Oxytetracycline, Florfenicol, and Tylosin
- Antiseptics: Savalon, Alcohol, Detergents, and Bleach
- Materials/equipment: protective clothing, vaccination and treatment syringe and needle; sprayer, cotton, gauze, face mask, gloves, laboratory supplies for collecting serum, blood, tissue and other samples.

Materials could be procured through public or private animal health service delivery posts/clinics. Mobile services to villages could be one efficient means of delivery of services.

Pre-requisites: The success of this intervention also relies on availability of the inputs and the service delivery system.

Action 1: Vaccination

Note:

- All calves must have one 3.5 L of colostrum within four to six hours of birth to receive adequate immunity. Calves that are not given enough antibodies at birth are at increased risk for pneumonia and scours throughout the entire growing period.

- Calf pneumonia is a major problem in dairy and beef herds. It is a multifactorial disease, and the most common post-mortem diagnosis in calves between one to five months of age. Agents of respiratory infection in calves include:
    - *Mannheimia haemolytica*, *Haemophilus somnus*, Infectious Bovine Rhinotracheitis (IBR), bovine Respiratory Syncytial Virus (RSV) and Parainfluenza III Virus (PI3), along with many other bacteria and mycoplasma species and viruses.
- Most calves that have pneumonia will have a fever and a rapid respiratory rate (over 60 per minute)

Do the following:

- Ensure transfer of passive immunity through adequate and timely provision of colostrum (**refer SOP for colostrum feeding**)
- Vaccinate all calves at 8 weeks of age for Pasteurellosis, IBR and if available parainfluenza.
- Vaccinate the breeding female 3-4 weeks prior to parturition to strengthen passive transfer of immunity through colostrum.
- Follow instruction by the manufacturer for vaccination (dose and site of injection) and for adoption refer details in ***Action 1 of Intervention 1 described for Diarrhea***.
- Don't inject needle in dirty area otherwise it might become infected
- Don't vaccinate pregnant cows/heifers in the first trimester (first 3 months of pregnancy)
- Don't vaccinate unhealthy and wet animals
- Don't wipe injection sites with disinfectant , this can reduce its effectiveness of the vaccine
- Don't give other medication at the time of vaccination
- Don't give Banamine or Flunix to a calf IM (give only IV) as it cause pain and severe muscle damage

Action 2: Isolation and quarantine

Note:

- Inadequate ventilation, crowding, commingling of animals from various farms (feedlot or sale barn situations), and other stresses have all been associated with pneumonia.
- Environmental factors include low environmental temperatures and high humidity and poor ventilation and also direct draughts onto calves themselves.

Do the followings:

- Separating the sick calf from the rest of the herd and provide sufficient attention to the sick calf; this prevent the spread of the diseases.
- Perform the following during isolation and quarantine of the pneumonic calf:
  - Avail a separate, dry and clean barn for isolating sick and newly introduced animals (newly purchased from other farms)
  - Properly care and seek professional help for treatment of isolated animals
  - Don't mix sick and apparently healthy animals in the isolation pen
  - Don't delay to seek professional help for cases of pneumonia
  - Don't treat sick young animals yourself if you are not an animal health professional

Action 3: Prevention and Treatment of sick animals

Note:

- A calf must grow one ~1/2 kg per day to maintain an adequate immune system.. Calves must have 5 to 7 Liters of milk per day, fresh water and starter feed to be healthy. Do not overlook the simple fact that calves must grow to be healthy, and in many situations we find inadequate nutrition to be a major cause of calf disease
- One cause of pneumonia in young calves is fluids going into the lungs via the windpipe (trachea). The first feeding of colostrum can cause problems if the

feeding rate is faster than swallowing rate. If colostrum is bottle fed it is important to use a nipple that matches the calf's ability to swallow.

Do the followings:

- Make sure colostrums is properly fed to the newborn if it is not suckling
- Viral and bacterial pneumonia spread fast between animals. Hence it is essential to immediately isolate and treat pneumonic calves
- Seek professional assistance if a calf shows signs of pneumonia (acute depression, lethargy and loss of appetite, separation from the remainder of the group, increased respiratory rate with an abdominal breathing and fever)
- Long acting oxytetracycline plus vitamins A, D, and E to all calves in the affected age group is the best antibiotic treatment
- Oxytetracycline is given at a dose rate of 10 mg/kg/day of non-long-acting product, or 20 mg/kg once of the long-acting product. Alternatively give Florfenicol (20 mg/kg, every 48 hr), or Tylosin (10–20 mg/kg, once to twice daily). Always read instruction for prescriptions before giving treatment
- Flunixin meglumine (1mg/kg IV every 24hrs) can be given in conjunction with antibiotic therapy for control of endotoxemia and inflammation.
- Therapy should continue for at least 72 hr after body temperature has returned to normal. Duration of treatment usually is 4–5 days.
- Whenever possible, treatment should be based on laboratory confirmation, especially in herd or flock outbreaks, when valuable animals are involved, or in acute or chronic cases when initial therapeutic attempts have failed
- Watch claves at the time of milk feeding for signs of pneumonia. Don't keep young animals without attention for too long if you see the signs of pneumonia
- Don't use antibiotics purchased from uncertified sources (forged drugs)
- Don't treat pneumonic animals yourself unless you are animal health professionals. Most drug resistance occurs due to unprofessional application of antibiotics

- Don’t give Banamine or Flunix to a calf IM (give only IV) as it cause pain and severe muscle damage

Action 4: Laboratory Confirmation of diseases causing agents

Note:

- Samples must be collected to confirm causative agents of diseases
- Appropriate samples for specific identification must be taken by veterinary health expert. A veterinarian caring for the sick individuals must always take samples from sick animals for the purpose of confirmatory diagnosis
- Samples must be properly labeled, kept in appropriate preservative or maintained in the proper cold chain until they reach laboratory.
- Samples should be stored locally only temporary until they are transported to the laboratory

Do the followings

- ✓ Collect at least 10 ml (2/3 of the tube) of blood from jugular vein in the neck region or tail using a vacutainer tube Clean the skin at the site of penetration using alcohol swab
- ✓ Always label each sample (date and identification code). Keep the tube slightly tilted at room temperature overnight (or stand for at least 2 hrs) to harvest serum if this is the purpose
- ✓ Serum samples should be decanted into a cryovial, labeled and kept in deep freezer at -21 ^{0}C.
- ✓ Collect about 5 g of fecal sample directly from the rectum using glove and keep in a universal container. Depending on the protocol, a preservative can be used for the feces
- ✓ Nasal swabs must be taken aseptically in a container designed for this.
- ✓ Follow instructions of the laboratory technologist for taking, storing and transporting other samples (***refer to SOP for laboratory procedures in identification of diseases causing agents***)

- ✓ Samples that require exporting should be temporarily stored at the National Animal Health and Diagnostic Center at Sebeta

3.3. REFERENCES/RESOURCES FOR FURTHER READING

- **Abraham, G., Reoder, D. T., and Roman, Z. 1992.** Agents associated with neonatal Diarrhea in Ethiopian dairy calves, *Trop. Anim. Health & Produ.* **25**: 239-248.
- **Heinrichs, A. J., andRadostits, O. M. 2001.**Health and production management of dairy calves and replacement heifers. In: Radositits, O. M. (ed): Herd Health, Food Animal Production Medicine, 3rd ed. Philadelphia, W. B. Saunders. Pp 333-395.
- **Ibrahim A. Lemma** A. 2009. Relationships between serum protein concentration and passive transfer of immunity, morbidity and mortality of dairy calves in market oriented urban dairy farms. Revue Méd. Vét., **160**, 8-9, 394-399
- **Jemberu, W. T. 2004.** Calf morbidity and mortality in dairy farms in Debrezeit and its environs, Ethiopia, Thesis for the Degree of Master of Science in Tropical Veterinary Epidemiology, Faculty of Veterinary Medicine, AAU. Derezeit, Ethiopia.
- **Johanson, J. M. and P. J. Berger. 2003.** Birth weight as a predictor of calving ease and perinatal mortality in Holstein cattle. J. Dairy Sci. 86:3745–3755
- **Lemma A. and Kebede S., 2011.** The effect of mating system and herd size on reproductive performance of dairy cows in market oriented urban dairy farms in and around Addis Ababa Revue Méd. Vét., **162**, 11, 526-530
- **Olsson, S. O., Viring, S., Emanuelsson, U., Jacobsson, S. O. 1993.** Calf disease and mortality in Swedish dairy herds. *Acta. vet. cand.* **34**: 263-269.
- **Rahmato, A. B. 2005.** An epidemiological study on major protozoal causes of calf diarrhea on selected dairy farms of central Ethiopia, FVM, AAU, DebreZeit, Ethiopia. Msc. Thesis in Tropical Veterinary Epidemiology.
- **Shiferaw, Y., Yohannes, A., Yilma, Y., Gebrewold, A. , and Gojjam, Y. 2002.** Dairy husbandry and health management at Holleta. Proceeding of the

16[th]conference of the Ethiopian veterinary association, Addis Ababa, Ethiopia, **Pp**: 103-119.

- **Waltner-Toews, D., Martin, S. W., Merk, A. H.1986b.** Dairy calf management, morbidity and mortality in Ontario Holstein herds. I. The data. *Preventive Veterinary Medicine,* **4**: 103-124.
- **AlemuYami. 2007.** How to make Urea Molasses Blocks (UMB) and feed to Sheep and Goats. Technical Bulletin No.1. Ethiopia Sheep and Goat productivity Improvement Program. R.C. Merkel (ed.). 10Pp.
- **FAO. 2011.** Rearing young ruminants on milk replacers and starter feeds. FAO Animal Production and Health Manual No. 13. Rome.
- **Lukuyu B, Gachuiri CK, Lukuyu MN, Lusweti C and Mwendia S (eds). 2012.** *Feeding dairy cattle in East Africa.* East Africa Dairy Development Project, Nairobi, Kenya. Pp.95.
- **Redeat Habtesellassie and Assefa Amalegegn. 2010.** Nutrition, Feeds and Feeding Management of dairy and Fattening Cattle Training Manual. Ed. C.P. Birkelo. ACDI/VOCA, Addis Ababa, Ethiopia.
